# Genome-wide identification of the *PFK* gene family and their expression analysis in *Quercus rubra*

**DOI:** 10.3389/fgene.2023.1289557

**Published:** 2023-11-09

**Authors:** Tae-Lim Kim, Hyemin Lim, Michael Immanuel Jesse Denison, Sathishkumar Natarajan, Changyoung Oh

**Affiliations:** ^1^ Department of Forest Bioresources, National Institute of Forest Science, Suwon, Republic of Korea; ^2^ 3BIGS Company Limited, Hwaseong, Republic of Korea

**Keywords:** gene expression pattern, gene family, genome-wide analysis, PFK, *Quercus rubra*

## Abstract

The glycolytic pathway involves phosphofructokinase (PFK), a rate-limiting enzyme that catalyzes the phosphorylation of fructose-6-phosphate. In plants, the two PFK members are ATP-dependent phosphofructokinase (PFK) and pyrophosphate-fructose-6-phosphate phosphotransferase (PFP). However, the functions of the PFK family members in *Quercus rubra* are not well understood. The purpose of this study was to investigate the genome-wide distribution of the PFK family members and their roles in *Q. rubra* by performing a systematic study of the phylogenetic relationships, molecular characteristics, motifs, chromosomal and subcellular locations, and *cis-*elements of *QrPFKs*. We identified 14 *QrPFK* genes in the genome of *Q. rubra*, followed by examining their expression in different tissues, including the roots, stems, and leaves. The phylogenetic tree divided the 14 *QrPFK* genes into two groups: 11 belonging to *PFK* and three belonging to *PFP*. The expression profiles of all 14 proteins were relatively the same in leaves but differed between stems and roots. Four genes (*Qurub.02G189400.1, Qurub.02G189400.2, Qurub.09G134300.1, and Qurub*.*09G134300.2*) were expressed at very low levels in both stems and roots, while two (*Qurub.05G235500.1 and Qurub.05G235500.1*) were expressed at low levels and the others showed relatively high expression in all tissues.

## 1 Introduction

Sugars are critical signaling molecules for the normal growth of higher plants. Trees produce sugars, including sucrose, glucose, and fructose, via photosynthesis. Glycolysis forms the basis for energy metabolism in many organisms. In plants, two types of phosphofructokinase (PFK) proteins exist that phosphorylate fructose-6-phosphate. ATP-dependent phosphofructokinase (PFK; EC 2.7.1.11) regulates the classical glycolytic pathway via glucose degradation (Plaxton, 1996). Chemically, sugars are a broad class of metabolites found primarily in the form of monosaccharides, such as glucose and fructose, or the disaccharide sucrose (Li et al., 2015). According to Siebers and Klenk (1998) (Siebers et al., 1998), PFK plays a key role in the conventional glycolytic pathway and is found in all three domains (ATP binding site, substrate-binding site, and the allosteric site) of organisms. PFK catalyzes the conversion of fructose-1,6-bisphosphate (F-1,6-BP) and fructose-6-phosphate (Fru-6-P). The evolution of different forms of PFK in plants is complex (Bapteste et al., 2003). The reversible phosphorylation of Fru-6-P to F-1,6-BP can also be catalyzed by pyrophosphate-fructose-6-phosphate phosphotransferase (PFP EC 1.7.1.90), another form of PFK, using pyrophosphate rather than ATP as a phosphoryl donor. PFP catalyzes this process and reaches near equilibrium in both directions, unlike PFK, which is nearly irreversible *in vivo*.

PFP has been well characterized at the molecular and biochemical levels in all three domains of life since the discovery of the first PFK in 1980 (Dunaway, 1983; Siebers et al., 1998; Mustroph et al., 2007; Darikova and Sherbakov, 2009; Zhu et al., 2013). PFP contains two distinct subunits, *α* and *β* (Cawood et al., 1988; Zhu et al., 2013; Qin et al., 2014). PFP has been the subject of extensive research, but PFK has received relatively little attention due to its instability when isolated during purification (Turner and Plaxton, 2003). Plant PFKs have been discovered in the plastids and cytosol of tomatoes (Isaac and Rhodes, 1982), *Triticum aestivum* (Mahajan and Singh, 1992), and *Ricinus communis* seeds (Podestá and Plaxton, 1994). The characteristics of PFKs from castor, wheat, potato tubers, and sprouting cucumber seeds were also examined (Cawood et al., 1988; Knowles et al., 1990; Mahajan and Singh, 1992; Teramoto et al., 2000). The evolution of sequencing technology over the last 10 years has enabled the sequencing of numerous plant genomes. Multiple other plant species have been reported to contain members of the PFK gene family, including poplar (Kim et al., 2023), rice (Mustroph et al., 2013), *Arabidopsis* (Mustroph et al., 2007), *saccharum* (Zhu et al., 2013), and spinach (Winkler et al., 2007). Gene duplication is a key process that provides novel evolutionary models of gene family expansion in eukaryotes (Ohno, 1970; Zhang, 2003). Genome research has provided concrete evidence that both large- and small-scale gene duplication events, including segmental and whole-genome duplication (WGD), are responsible for the origin of gene family members. According to Jiao et al. (2011), all five species of the family Rosaceae have undergone at least one WGD (Velasco et al., 2010; Shulaev et al., 2011; Zhang et al., 2012; Initiative et al., 2013; Wu et al., 2013). Recent sequencing of the pear genome (Wu et al., 2013) and gene family analyses have revealed that WGD is a major factor in gene family expansion (Li et al., 2015; Qiao et al., 2015; Li et al., 2017). Therefore, gene families can grow because of large-scale duplication events, such as WGD or segmental duplication.

Recently, studies on *PFK* gene family function under abiotic stress have been reported. The expression of PFP subunit genes in *Arabidopsis* seedlings increased under salt and osmotic stresses, indicating a role for PFP in their adaptation to these stresses (Lim et al., 2014). There are studies showing that the *PFK* gene family is involved in response to hypoxia. For example, Angelika Mustroph reported changes in the transcript levels of *PFK* and *PFP* in rice and slight changes in enzyme activity under hypoxia (Mustroph et al., 2013). Additionally, increased gene expression of the PFP enzyme was confirmed in maize leaves under hypoxia due to extreme waterlogging (Panozzo et al., 2019). In cassava, the expression patterns of *MePFK*s across various developmental stages, organs, and under waterlogging stress highlighted the significant role of *MePFPA1* in cassava growth and development. Furthermore, it was observed that pyrophosphate (PPi)-dependent glycolysis bypass was enhanced in cassava under waterlogging stress (Wang et al., 2021). In *Gossypium barbadense*, a co-expression network analysis of 26 genes from *GhPFK* using RNA-seq data revealed *GhPFK11* and *GhPFK17* as common hub genes. These genes are potential candidates associated with drought stress tolerance (Mehari et al., 2022).

Northern red oak (*Q. rubra* L.) is an ecologically and economically important forest tree distributed in North America and southeastern Canada at an altitude of 1,700 m or higher (Godfrey, 1988). It has a straight trunk, grows as a large hardwood, has strong cold resistance, and can grow even below −40°C; therefore, it is used as a floorboard and building material in North America (Sander, 1990) and is reported to be resistant to stress such as drought or frost (Straigyte and Zalkauskas, 2012). Oak is a diploid forest tree with a relatively manageable genome size (approximately 600–800 Mb) and a haploid chromosome number of 12 (Kremer et al., 2007; Chen et al., 2014). Native to the Northern Hemisphere, the genus *Quercus* includes deciduous and evergreen tree species that grow at latitudes ranging from cool temperate to tropical regions throughout America, Asia, Europe, and North Africa. Most oak species are found in North America, with approximately 90 species in the United States and 160 in Mexico, of which 109 are native species. *Q. rubra* is a tree species of interest as it is currently expanding its habitat as an alternative plantation species in Korea. In this study, we used *Q. rubra* as the research material. It is a key species for woody plant research and has a genome sequence with a clear genetic background. However, studies on the PFK family in *Q. rubra* are lacking. Therefore, we conducted a study on the *PFK* gene of *Q. rubra* to establish selection indices for trees with excellent traits and to improve varieties.

In this study, the *Q. rubra* genome database was used to identify *PFK* gene family members. Gene structure, chromosome distribution, protein subcellular localization, promoter *cis-*acting elements, synteny, and expression of *PFK* genes in different tissues of *Q. rubra* were determined using real-time quantitative (qRT-PCR). The results provide a theoretical basis for future research on the functions of the *QrPFK* genes.

## 2 Materials and methods

### 2.1 Plant materials

Nine-year-old *Q. rubra* served as the experimental subject. At the National Institute of Forest Science in Korea (37° 15′04″ N, 136° 57′59″ E), these trees were planted. In 2014, (1-0) seedlings were planted in Hwaseong-si, Gyeonggi-do using seeds introduced from Michigan, United States. We collected samples in June 2023, when daytime temperatures were 23°C–28°C. Leaves, stems grown last year, stems newly grown this year, and root tissues were collected from each of the three trees and used for RNA extraction and protein expression analysis. For the extraction of RNA and protein later, fresh tissue samples were taken from *Q. rubra* trees, instantly frozen in liquid nitrogen, and stored at −80°C.

### 2.2 Identification, physiochemical characteristics, and subcellular localization of PFKs in *Quercus rubra*

To perform genome-wide analysis in *Q. rubra*, genome data (Qrubra_687_v2.1) were retrieved from Phytozome version V13 (https://phytozome-next.jgi.doe.gov/info/Qrubra_v2_1 accessed 26 June 2023). The hidden Markov model (HMM) profile corresponding to the PFK domain (PF00365; (Wegener and Krause, 2002)) from the Pfam protein family database was used to scan identified proteins in the *Q. rubra* genome (Q. rubra_v2.1) using HMMERv3 (Eddy, 2009). The PFK family protein sequences were aligned using the HMM model in HMMERv3. Putative PFK genes were verified by searching the NCBI Conserved Domain Database (CDD, accessed 26 June 2023) to confirm the presence of the conserved PFK domain. The GTF file of the *Q. rubra* genome was retrieved from the Phytozome website. Based on the annotation file and the gene IDs of the *Q. rubra PFK* gene family members, the chromosome mapping maps were drawn by the TBtools software (Chen et al., 2020). The physicochemical properties, such as molecular weight, isoelectric point, aliphatic index, and grand average of hydropathy (GRAVY), were retrieved from Expasy ProtParam (https://web.expasy.org/protparam/; accessed on 26 June 2023). The subcellular localization of the QrPFK proteins was predicted Using WoLF PSORT (https://wolfpsort.hgc.jp/; accessed 27 June 2023) (Wang et al., 2021). Based on sorting signals, amino acid composition, and functional patterns, such as DNA-binding motifs, the amino acid sequences of the proteins were translated into numerical localization features. GraphPad Prism 9 was used to convert the protein localization cues at a specific subcellular level into a heatmap representation.

### 2.3 Multiple sequence alignment analysis and phylogenetic relationship analysis of *QrPFKs*

The protein sequences of the QrPFK family collected from the Phytozome V13 database were aligned using ClustalW (Thompson et al., 2003) (https://www.genome.jp/tools-bin/clustalw, accessed 26 June 2023). The *.aln file from ClustalW was loaded into ClustalO (Sievers and Higgins, 2014) through command-line execution. ClustalO produces a distance matrix that illustrates the amino acid identity. The *.dnd file obtained from ClustalW was loaded onto the iTOL server (accessed on 26 June 2023) (Letunic and Bork, 2007) to construct a phylogenetic tree. The phylogenetic tree was constructed using neighbour-joining algorithm with 1,000 bootstrap replicates. Using the hmm file of the PFAM model of PFK domain proteins, the PFK family proteins were extracted from *Populus deltoides, Arabidopsis thaliana, R. communis, Solanum tuberosum,* and *Oryza sativa*. The PFK protein sequences from all species, including *Q. rubra,* were aligned using CLUSTALW (https://www.genome.jp/tools-bin/clustalw; accessed on 27 June 2023) and the iTOL server was used for phylogenetic tree construction (https://itol.embl.de/, accessed on 27 June 2023). PdPFKs, AtPFKs, StPFKs, RcPFKs, and OsPFKs were classified as PFKs and PFPs based on the previously reported classification of the corresponding genome assemblies.

### 2.4 Gene structure analysis and motif location analysis of the *QrPFKs*

In the Gene Structure Display Server (GSDS) (Hu et al., 2015), both the coding sequence (CDS) and genomic sequences corresponding to *QrPFK*, the exon-intron structure of *QrPFK* genes was graphically presented. We predicted the conserved motifs in the *QrPFK* gene family with an e-value of 10^–5^ in *Q. rubra* using MEME (http://meme-suite.org/) and identified a total of 10 conserved motifs. Finally, TBtools was used to examine the gene structure and motifs of *QrPFK* genes (Chen et al., 2020).

### 2.5 Chromosomal mapping, gene duplication, collinearity and synteny analysis of *QrPFKs*

The genome and annotations for *P*. *deltoides*, *A*. *thaliana*, *S*. *tuberosum*, *R. communis, and O. sativa* were downloaded for comparative studies. *P. deltoides* genome was downloaded from Phytozome V13 (Kim et al., 2023); *A. thaliana* genome at chromosome level was downloaded from TAIR v10.1 (https://www.arabidopsis.org/), *S. tuberosum* from Ensembl Plants (http://plants.ensembl.org/Solanum_tuberosum/Info/Index), *R. communis* from Oil Plant Database (http://oilplants.iflora.cn/), and *O. sativa* from IRGSP-1.0 genome available at NCBI (https://www.ncbi.nlm.nih.gov/datasets/genome/GCF_001433935.1/). Tandem and segmental duplications mediate the development and progression of plant genomes by promoting new members of a gene family. Segmental duplications cause gene duplication via polyploidy, whereas tandem duplications are caused by the crossover of shorter pieces (Cui et al., 2007; Liu et al., 2014). We used BLASTP searches to examine tandem and segmental gene duplications in *PFKs*. The ‘MCScanX’ function of the TBtools software with default parameters was used to predict gene duplications of *QrPFK* genes. The MCScanX Diamond output was used to calculate the replication events of the *Q. rubra* genome. The Duplicate_gene_classifier program in MCScanX (https://github.com/wyp1125/MCScanX, accessed on 26 June 2023) was used to examine the duplication type of each *QrPFK* gene. A simple Ka/Ks calculator in TBTools (Chen et al., 2020) was used to calculate the Ka/Ks ratio of tandem repeat gene pairs in the *QrPFK* genes. Additionally, the Advanced Circos function of TBtools software (version 1.130) was used to visualize WGD or segment duplications. Furthermore, the synteny of *QrPFK* genes with the *PFK* genes of *P. deltoides*, *A. thaliana*, *O. sativa*, *R. communis*, and *S. tuberosum* was visualized using the One-Step MCScanX function of the TBtools software. The Dual Synteny Plot for the MCScanX function of the TBtools software (version 1.130) was used to visualize the synteny. Pairs of segmental duplications were used to estimate Ka, Ks, and their ratios. The Ka and Ks values for each pair were calculated using TBtools in accordance with the YN model (Yang and Nielsen, 2000).

### 2.6 *Cis-*acting regulatory elements (CREs)

The 1.5 kbp upstream promoter sequence of the *QrPFK* genes of *Q. rubra* was retrieved using the Phytozome v13 database (https://phytozome.jgi.doe.gov/pz/portal.html) (Goodstein et al., 2012) to examine the promoter regions. The PlantCARE database (http://bioinformatics.psb.ugent.be/webtools/plantcare/html/, accessed on 28 June 2023) (Lescot et al., 2002) was used to identify CREs, enhancers, and repressors. Toolkit Biologists Tools (TBtools) software was used to graphically represent the positions of CREs in the sequences.

### 2.7 Three-dimensional molecular modeling and structural validation of PFK proteins

The secondary structures of QrPFK were predicted using SOPMA tool (https://npsa-prabi.ibcp.fr/cgi-bin/npsa_automat.pl?page=/NPSA/npsa_sopma.html, accessed on 25 September 2023). The three-dimensional protein structures of all the QrPFK proteins were constructed through homology approach and validated through Ramachandran plot using Swiss Model webserver (https://swissmodel.expasy.org/, accessed on 25 September 2023).

### 2.8 Protein interaction network and KEGG enrichment analysis

Proteins in plant systems tend to interact with each other, regardless of their classes or different groups, to regulate different signaling pathways. To evaluate the interactions of QrPFK proteins with other interactors, the protein sequences of the selected QrPFK proteins were used in the STRING tool (Search Tool for the Retrieval of Interacting Genes; https://string-db.org/, accessed on 25 September 2023) (Szklarczyk et al., 2019)) for constructing interactive networks with respect to their ortholog pairs in *Populus*. The confidence threshold criterion was set at 0.40, and a maximum of 10 interactors were selected in the first shell. STRING database offers functional annotation of the mapped proteins against the Kyoto Encyclopedia of Genes and Genomes (KEGG) (https://www.genome.jp/kegg/) database.

### 2.9 RNA extraction and qRT-PCR

The Beniprep^®^ Super Plant RNA extraction kit (InVirusTech Co., Gwangju, Korea) was used to extract total RNA from *Q. rubra* tissues. RNA was extracted from three biological replicates. RNA samples were reverse-transcribed into single-stranded cDNA using cDNA EcoDryTM Premix (TaKaRa, Shiga, Japan). Real-time qPCR was performed using the CFX96 Touch Real-Time PCR Detection System (Bio-Rad, Hercules, CA, United States) with IQtm SYBR Green Supermix (Bio-Rad). The reaction consisted of the following steps: one cycle at 95°C for 3 min, followed by 36 cycles at 95°C for 15 s and 60°C for 60 s. The 2^−ΔΔCt^ method was used to analyze relative transcript abundance (Livak and Schmittgen, 2001). The expression levels of *α-tub* and *18S rRNA* were used to normalize the qPCR results (Makela et al., 2016; Migueal et al., 2017). The gene-specific primers used are listed in Supplementary Table S1.

### 2.10 Measurement of phosphofructokinase content

A PFK ELISA kit (MyBioSource, San Diego, CA, United States) was used to determine the amount of PFK in homogenized leaf, stem, and root samples. All procedures were performed according to the manufacturer’s instructions. Absorbance was measured at 450 nm using an automatic plate reader (SpectraMax M2, Molecular Devices, San Jose, CA, United States).

### 2.11 Statistical analysis

For the qPCR and level of PFK protein analyses, a one-way ANOVA was used, and for multiple comparisons, the Tukey test for honestly significant differences (HSD) was applied. The level of statistical significance was set at *p* < 0.05. Outcomes were presented as means ± standard deviations (SD).

### 2.12 Schematic diagram of the methodorolgy

A flow chart of the overall methodology is shown in Supplementary Figure S1.

## 3 Results

### 3.1 Identification of PFKs, physiochemical characteristics, and subcellular localization

**FIGURE 1:**
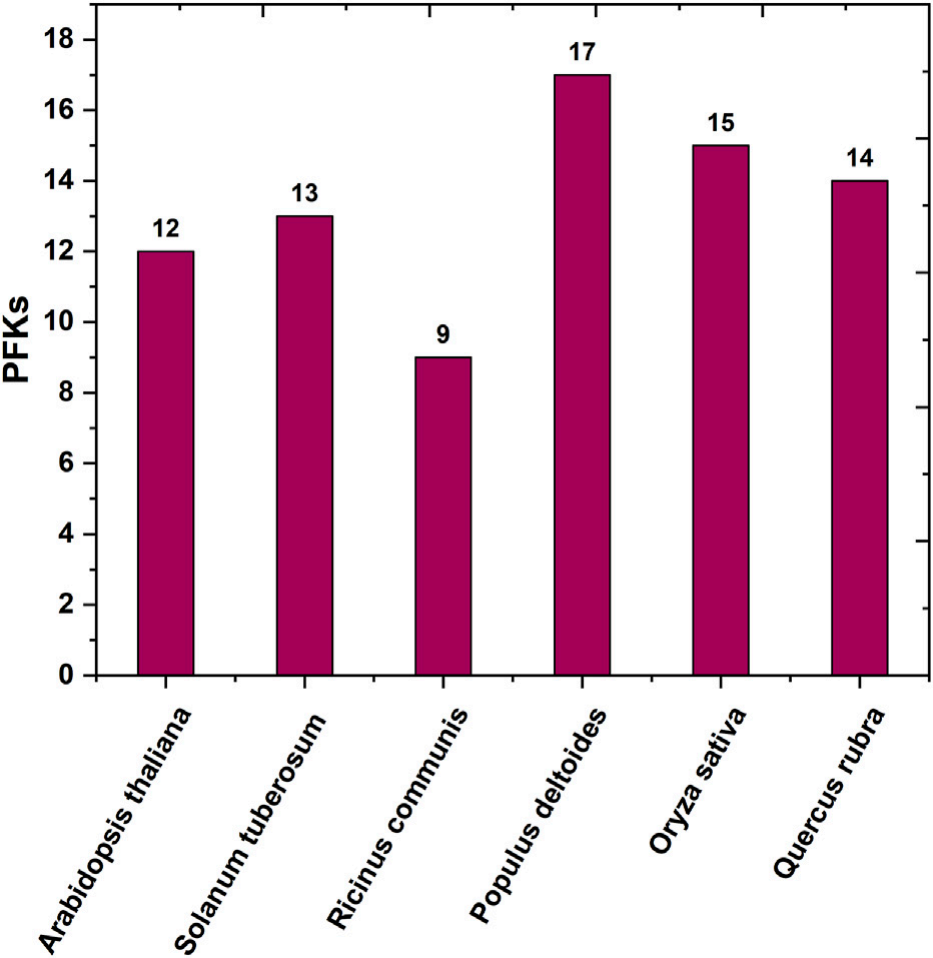
Bar graph of the number of protein-coding *PFK* gene family across different species reported earlier.

**FIGURE 2:**
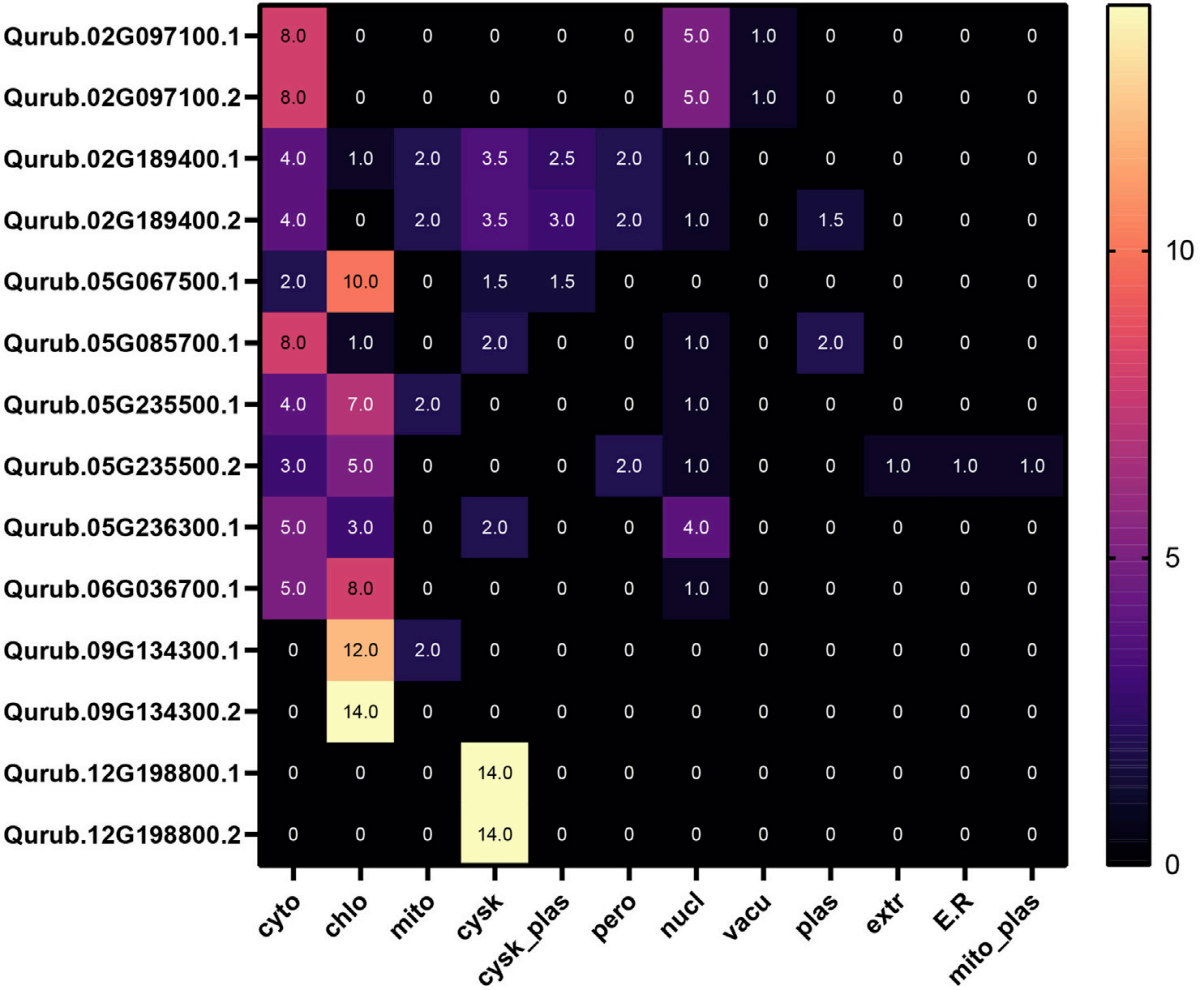
Subcellular localization of QrPFK proteins.

We included all the 14 *PFK* genes identified through HMMSearch in the *Q. rubra* genome for further analysis (Figure 1). The domains of PFK proteins were predicted using a conserved domain database. All 14 proteins contained domains predicted using the HMMer3 approach. The proteins detected were subjected to physicochemical characterization using ProtParam. The main parameters, such as the molecular weight, isoelectric point, aliphatic index, and GRAVY, are listed in Table 1. The subcellular localization of QrPFKs is shown in Figure 2. Among the QrPFK proteins, Qurub.05G236300.1 had a lower molecular weight (33.55 KDa), whereas Qurub.02G097100.1 and Qurub.02G097100.2 had higher molecular weights (67.31 KDa). The calculated pI values for Qurub.05G067500.1, Qurub.05G085700.1, Qurub.05G236300.1, Qurub.05G235500.1, Qurub.12G198800.2, Qurub.12G198800.1, Qurub.05G235500.2, and Qurub.02G189400.1 (pI < 7) indicated their acidic nature, while those of Qurub.02G097100.1, Qurub.02G097100.2, Qurub.09G134300.2, Qurub.06G036700.1, and Qurub.02G189400.2 (pI > 7) implied their alkaline nature. Qurub.05G085700.1 showed a lower aliphatic index, while Qurub.02G097100.1 and Qurub.02G097100.2 demonstrated higher aliphatic indices. Qurub.05G235500.1 and Qurub.05G235500.2 had very low GRAVY indices. Aliphatic index proteins are highly stable proteins at higher temperatures whereas negative GRAVY denotes that these proteins are hydrophilic in nature. Estimation of subcellular localization of QrPFK proteins using the WoLF PSORT tool (https://wolfpsort.hgc.jp/) (Horton et al., 2007) revealed that most of them are localized in the cytoplasm, chloroplasts, and cytoskeleton. Particularly, QrPFK family members Qurub.12G198800.1 and Qurub.12G198800.2 are localized in the cytoskeleton. The abundance of QrPFKs is represented as a heat map in Figure 2.

**TABLE 1:** Physicochemical properties of PFK proteins in *Quercus rubra.*

Protein	Class	Chromosome	Start	End	Aliphatic index	Protein length (aa)	Molecular weight (KDa)	Theoretical pI	GRAVY
Qurub.02G097100.1	QrPFPα	LG2	15812091	15819256	2,678	615	67.31	7.20	−0.085
Qurub.02G097100.2	QrPFPα	LG2	15812093	15819225	2,394	615	67.31	7.20	−0.085
Qurub.02G189400.1	QrPFK	LG2	37049077	37054733	2079	502	55.15	6.74	−0.249
Qurub.02G189400.2	QrPFK	LG2	37049077	37054733	2083	439	47.81	8.05	−0.195
Qurub.05G067500.1	QrPFPβ	LG5	14071785	14080918	2,291	569	62.10	6.01	−0.127
Qurub.05G085700.1	QrPFK	LG5	17838758	17843662	1965	479	52.98	6.02	−0.244
Qurub.05G235500.1	QrPFK	LG5	57134456	57140669	1947	564	62.28	6.43	−0.333
Qurub.05G235500.2	QrPFK	LG5	57134455	57140670	1977	510	56.64	6.69	−0.286
Qurub.05G236300.1	QrPFK	LG5	57322593	57326919	1,639	305	33.55	6.41	−0.177
Qurub.06G036700.1	QrPFK	LG6	6177636	6187694	2,222	537	58.75	7.93	−0.080
Qurub.09G134300.1	QrPFK	LG9	25861848	25872699	2,587	533	58.97	7.05	−0.244
Qurub.09G134300.2	QrPFK	LG9	25864776	25872699	1844	436	48.38	7.72	−0.234
Qurub.12G198800.1	QrPFK	LG12	36918161	36926523	2,192	484	52.98	6.68	−0.169
Qurub.12G198800.2	QrPFK	LG12	36918124	36926572	2,272	482	52.79	6.68	−0.176

Abbreviations: LG, linkage group; pI, isoelectric point; GRAVY, grand average of hydropathy.

### 3.2 Multiple sequence alignment analysis and phylogenetic relationship analysis of *QrPFKs*

**FIGURE 3:**
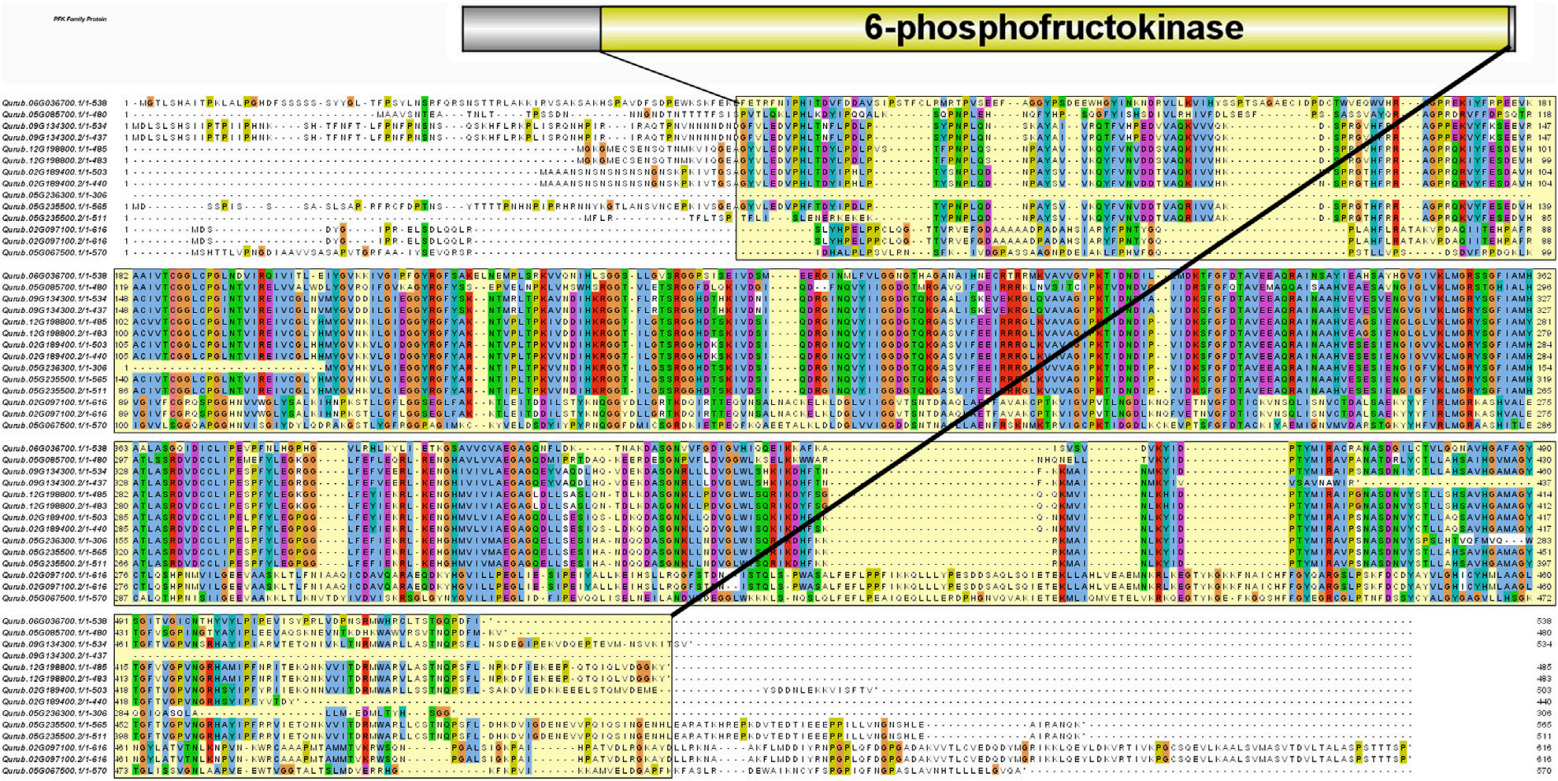
Protein sequence alignment performed with ClustalW. The yellow highlighted region represents the 6-phosphofructokinase domain.

**FIGURE 4:**
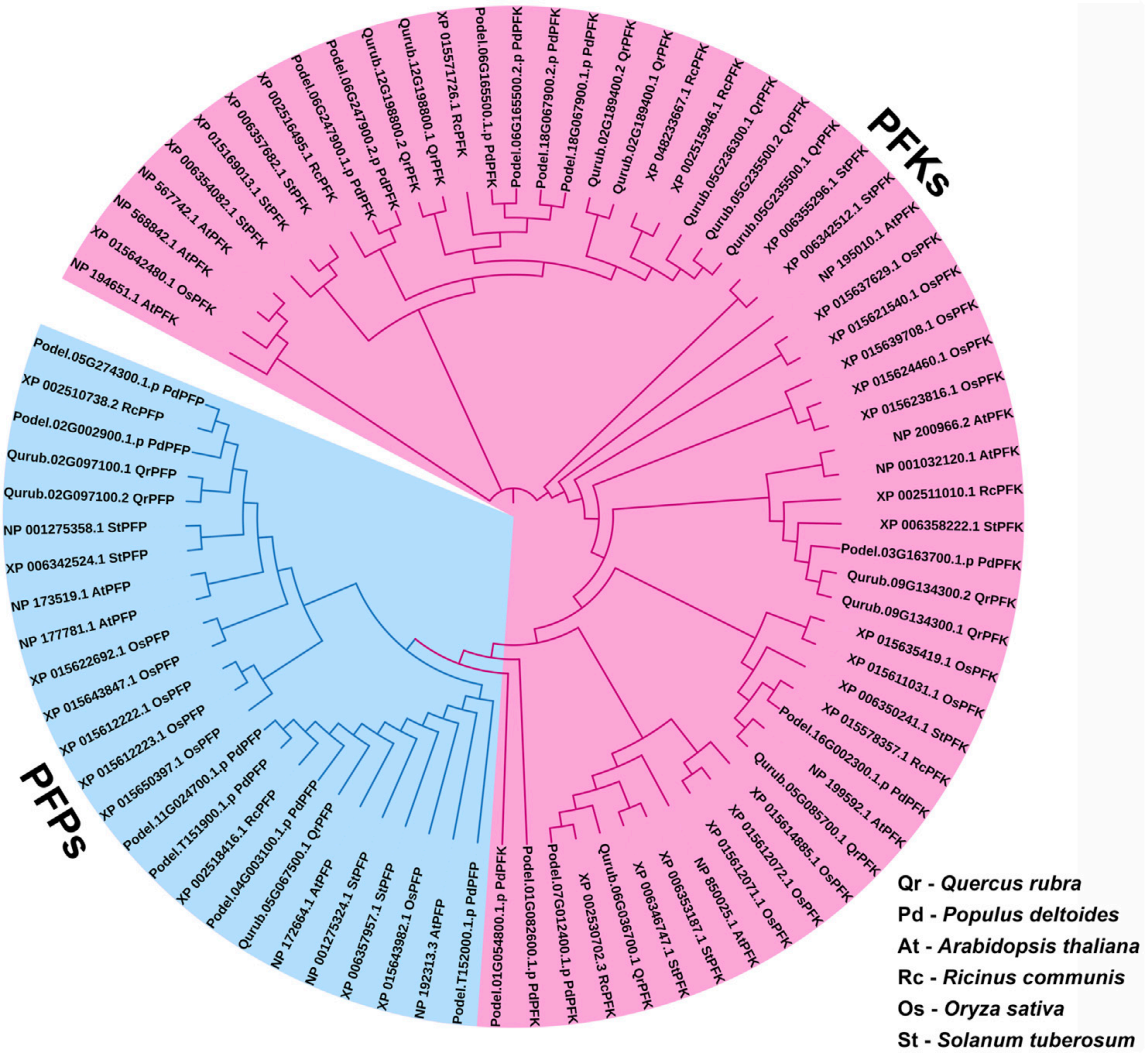
Phylogenetic tree of PFK gene family proteins identified in *Quercus rubra*, related tree species *Populus deltoides*, *A. thaliana*, *Ricinus communis*, *Oryza sativa*, and *S. tuberosum*.

A total of 14 protein sequences were collected using the PFAM-based HMM search algorithm. Among the aligned sequences of all 14 proteins based on the six PFK domains using ClustalW, sequence variations were observed in the sequences of three proteins: Qurub.05G067500.1, Qurub.02G097100.1, and Qurub.02G097100.2 (Figure 3 and Supplementary Figure S2). Protein sequence identity testing among the 14 proteins containing a PFK domain revealed that Qurub.05G067500.1 is identical to two other proteins, Qurub.02G097100.1 and Qurub.02G097100.2. These three proteins (Qurub.05G067500.1, Qurub.02G097100.1, and Qurub.02G097100.2) shared less sequence identity with other proteins containing the PFK domain. To completely understand the nature of these three proteins, all members of the PFK superfamily from model plant species, such as *P. deltoides*, *A. thaliana*, *O. sativa*, *R. communis*, and *S. tuberosum*, were downloaded and renamed based on their annotations as PFKs and PFPs. The phylogenetic tree constructed using the iTOL web server was represented in the form of a circular tree (Figure 4). Three variable proteins (Qurub.05G067500.1, Qurub.02G097100.1, and Qurub.02G097100.2) were grouped with the PFPs of other species, whereas the remaining proteins were grouped with their PFKs. All PFKs are shown in pink, and PFPs are shown in blue.

### 3.3 Gene structure analysis and motif location analysis of the *QrPFKs*

**FIGURE 5:**
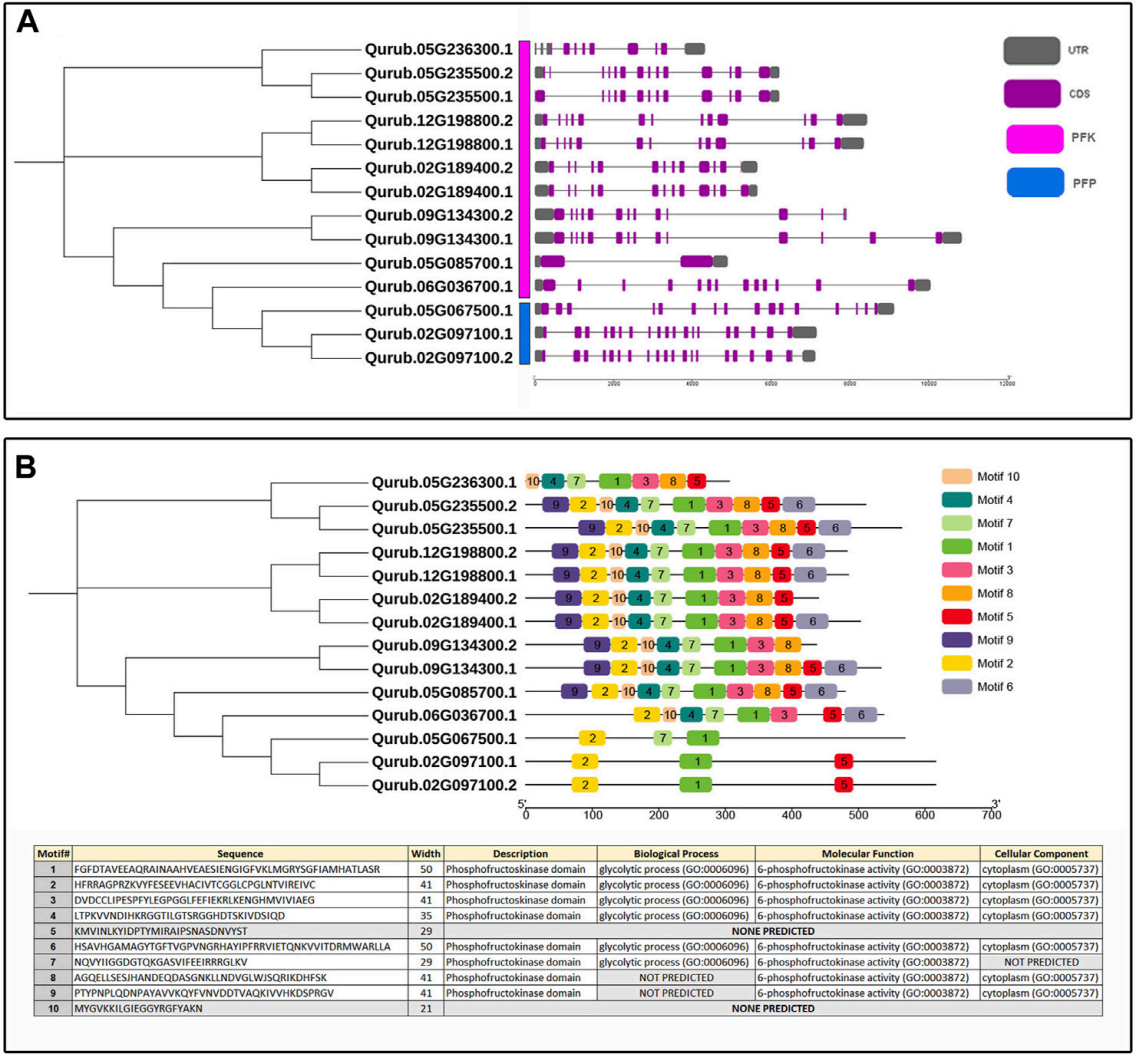
**(A)** Exon-intron structure of *QrPFK* genes; **(B)** Phylogenetic tree based on conserved motifs in *QrPFKs*. The phylogenetic tree was constructed based on the full-length sequences of QrPFK proteins.

The exon-intron structures were also investigated according to the location of the exon and intron structures in the *Q. rubra* genome (Figure 5A). The results showed that the genes in the same phylogenetic clades tended to share the same or similar exon/intron structures. Totally, there are 1–18 introns in the *QrPFK* gene family. There are 18 introns in the *QrPFP* members, *Qurub.02G097100.1* and *Qurub.02G097100.2* while 15 introns in *Qurub.05G067500.1*. There are only 1–12 introns in the studied *QrPFK* members. In general, we discovered that *QrPFK*s had fewer introns than *QrPFP*s—more than 15 introns (Figure 5A). Ten motifs were identified in *QrPFK*s (Figure 5B). Motifs 1 and 2 are conserved in all *QrPFK*s. Motif 5 was not present in Qurub.05G067500.1; motif 7 was an exception. As shown in Figure 5B, motifs 5 and 7 are of equal length. Motif 5 appeared to be less functional than motif 7, as none of the GO terms were predicted for motif 5. Motifs 1, 2, 3, 4, and 6 appeared functionally active, with GO term annotations specific to 6-PFK activity. As a result, motifs 1 and 2 were considered conserved functional domains in all QrPFKs.

### 3.4 Chromosomal localization, collinearity and synteny analysis of *QrPFKs*

**FIGURE 6:**
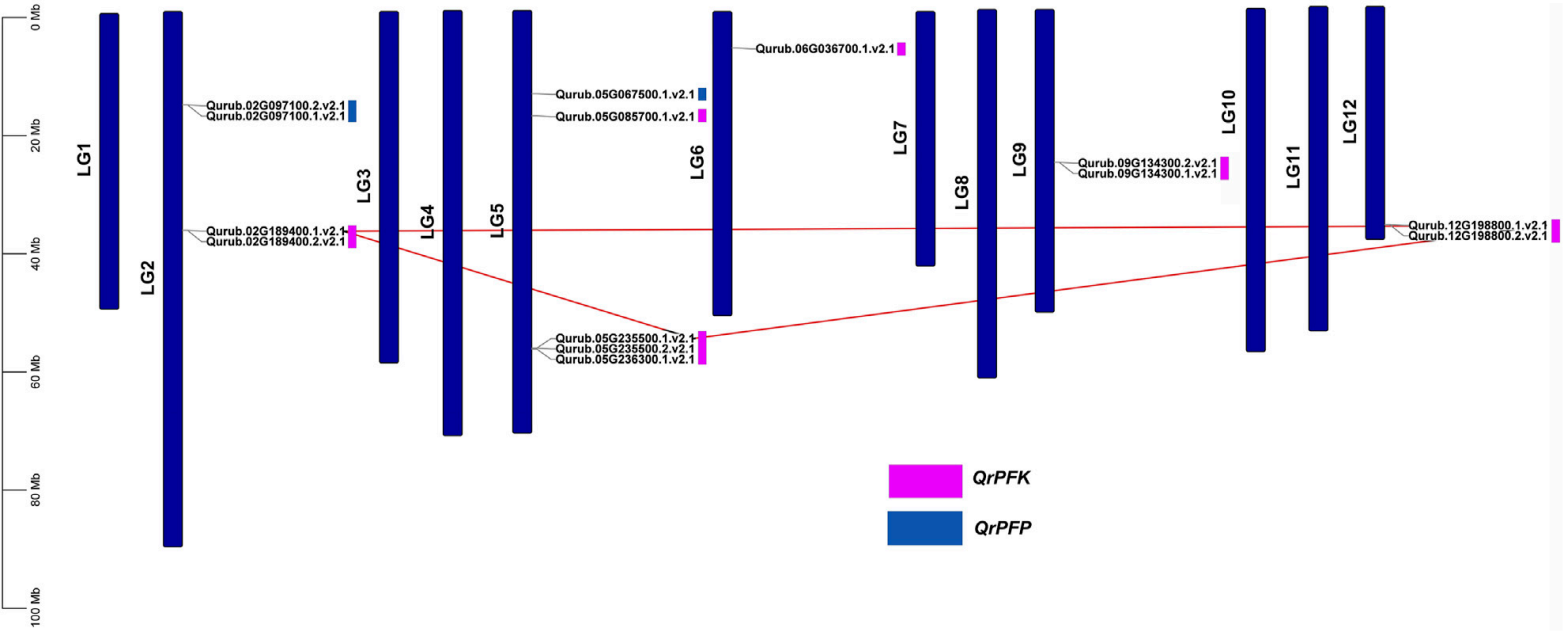
Chromosomal map of *Quercus rubra* with the distribution of the *QrPFK* genes. The left scale indicates the size of each chromosome.

**FIGURE 7:**
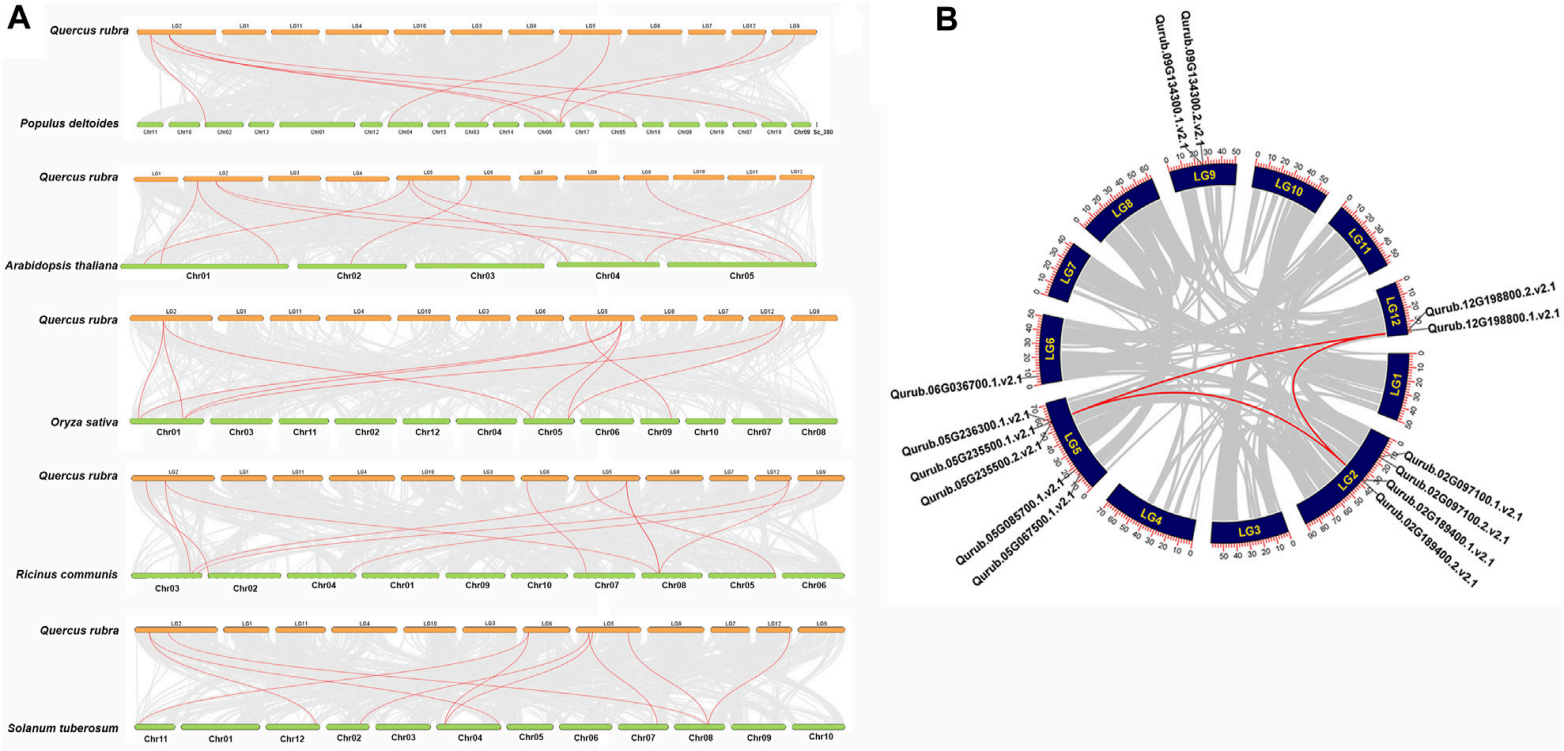
Synteny and collinearity analysis *QrPFK* gene family. **(A)** Interspecific collinearity relationship between *QrPFK* genes and *PFKs* from *Populus deltoides*, *A. thaliana*, *Oryza sativa*, *Ricinus communis*, and *S. tuberosum*. The chromosomes of *Quercus rubra* are represented in orange (chromosome numbers marked above the bars), while those of *Populus deltoides*, *A. thaliana*, *Oryza sativa*, *Ricinus communis*, and *S. tuberosum* are represented in parrot green (chromosome numbers marked below the bars). The red line represents the homologous gene pairs and the grey line represents the collinearity between respective genomes. **(B)** The collinear relationship among the *PFK* genes of different species and the *QrPFK* genes. The red line represents the segmental duplication and the grey line represents the collinearity between the same genome.

The localization of the *PFK* genes along the chromosomes of *Q.rubra* is shown in Figure 6. *QrPFK* family members are partially distributed and scattered, with 14 genes distributed on five *Quercus* chromosomes, mostly relatively close to the telomeric region. Qurub.02G189400.1, Qurub.02G189400.2, Qurub.09G134300.1, and Qurub.09G134300.2 are located outside telomeres. Of the 14 members of the *QrPFK* family, most genes were mapped to LG02 and LG05, whereas the least abundance was observed in LG06. All the *QrPFPs* are in LG02 and LG05. Collinearity analysis was performed using PFK family members from *Q. rubra*, *A. thaliana*, *O. sativa*, *R. communis*, and *S. tuberosum* to ascertain the collinear connections of the 14 *QrPFK* genes among different species (Figure 7). *PFKs* from *Arabidopsis* and castor showed collinearity with *QrPFK* members on *Quercus* chromosomes LG2, LG5, LG6, LG9, and LG12. Rice *PFKs* showed collinearity with *QrPFK* members in *Quercus* chromosomes LG2, LG5, and LG12, whereas potato *PFKs* showed collinearity with *QrPFK* members in *Quercus* chromosomes LG2, LG5, LG6, and LG12. The ratio of nonsynonymous (Ka) to synonymous (Ks) substitution rates is a critical parameter for inferring evolutionary dynamics after gene duplication. A Ka/Ks value of 1 indicates neutral selection, <1 indicates negative selection, and >1 indicates positive selection (Hurst, 2002). To calculate the evolutionary time of *QrPFKs*, we analyzed the Ka/Ks indices for duplicate gene pairs (paralogs) (Table 2). The Ka/Ks ratios for the *PFK* gene pairs ranged from 0.10 to 0.15, with an average of 0.12 (Table 2). The three paralogs had low Ka/Ks ratios (*p* < 0.3). Therefore, most PFK paralogs may have been subject to strong purifying or stabilizing selection and have the shortest divergence time (7.30 million years ago (Mya)), indicating that they may have retained their function after duplication. Estimation of the divergence time for three *PFK* paralog pairs revealed that gene duplication occurred between 8.3 and 9.6 Mya, implying that the spread of these paralogs occurred during the last round of *Quercus* WGD.

**TABLE 2:** Ka/Ks-ratio values of the paralog *QrPFK* pairs.

Gene 1	Gene 2	Ka	Ks	Ka_Ks	Time (Mya^a^)
Qurub.12G198800.1.v2.1	Qurub.02G189400.1.v2.1	0.126747622	0.859822622	0.14741136	9.660642
Qurub.12G198800.1.v2.1	Qurub.05G235500.1.v2.1	0.119535704	1.035450036	0.115443237	9.110953
Qurub.02G189400.1.v2.1	Qurub.05G235500.1.v2.1	0.10937211	1.045139359	0.104648351	8.336289

^a^ Million years ago.

### 3.5 *Cis-*acting regulatory elements (CREs)

**FIGURE 8:**
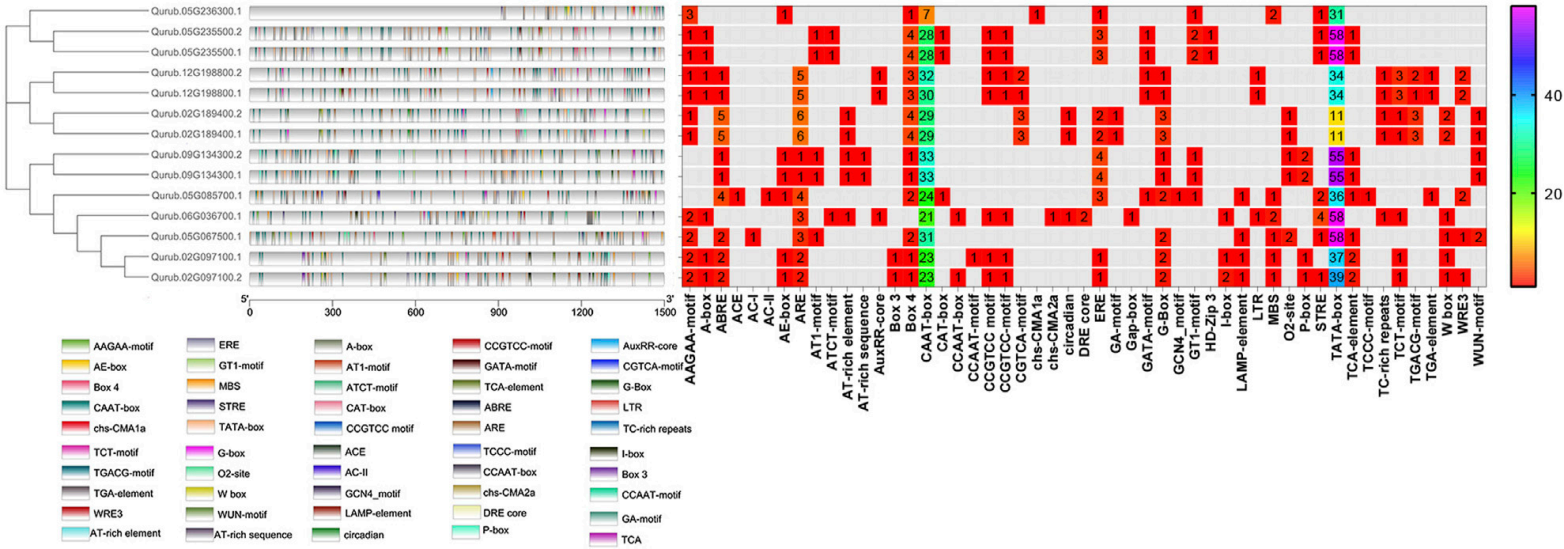
Frequency distribution of CREs in 14 *QrPFKs*. They were visualized using the Simple BioSequence Viewer in TBtools software. Different gradient color bars along the chromosome length indicate different types of motifs. The frequency distribution is represented using spectral colors ranging from 1 to 58.

The PlantCARE program was applied to a 1,500 bp upstream region of 14 *QrPFK* genes to analyze 32 types of putative CREs and divide them into three main types based on their functional annotation: phytohormone response, plant growth and development, and stress response in *Q. rubra*. The presence of the TATA-box, CAAT-box, AT-TATA-box, A-box, and TATA elements in the binding site was investigated. The overall frequency distribution of *PFK* and *PFP* CREs in *Q. rubra* is documented in Supplementary Table S2. The TATA-box and CAAT-box are abundant in all 14 *QrPFK* genes and initiate transcription by acting as binding sites for transcription factors, while the TATA-element helps bind the TATA-binding proteins (TBP) at the initiation site. The TATA-box and A-box are promoter binding sites (Figure 8). In the *QrPFK* promoters, 1,595 CREs were identified, of which the CREs for the stress response were the most enriched. Particularly, *QrPFK* genes were highly enriched in the 60 K protein binding site, *cis-*acting elements involved in the salicylic acid and abscisic acid reactions, the *cis-*acting regulatory element involved in the MeJA responsiveness, *cis-*acting regulatory elements essential for anaerobic induction and involved in the light reaction and regulation of zein metabolism, common *cis-*acting element in promoter and enhancer regions, core promoter element around −30 of transcription start, MYB binding sites involved in dryness inducibility and light reactivity, MYBHv1 binding site, short_function, light responsive element, and parts of a conserved DNA module involved in light responsiveness and light responsive element (Supplementary Table S2). Critical regulatory elements, the TATA box and CAAT box, as well as other regulatory elements, such as ARE, ERE, ABRE, STRE, MRE, and WRE3, were more abundant in *QrPFK* genes than in *QrPFP* genes. Notably, the involvement of CGTCA and TGACG motifs in MeJA responsiveness was observed for *QrPFKs* but not for *QrPFPs*. Most *cis-*regulatory elements were observed to be light-responsive elements. The frequency distribution of CREs in each gene family member is shown in Figure 8, with a color interpretation.

### 3.6 Three-dimensional molecular modeling and structural validation of PFK proteins

**FIGURE 9:**
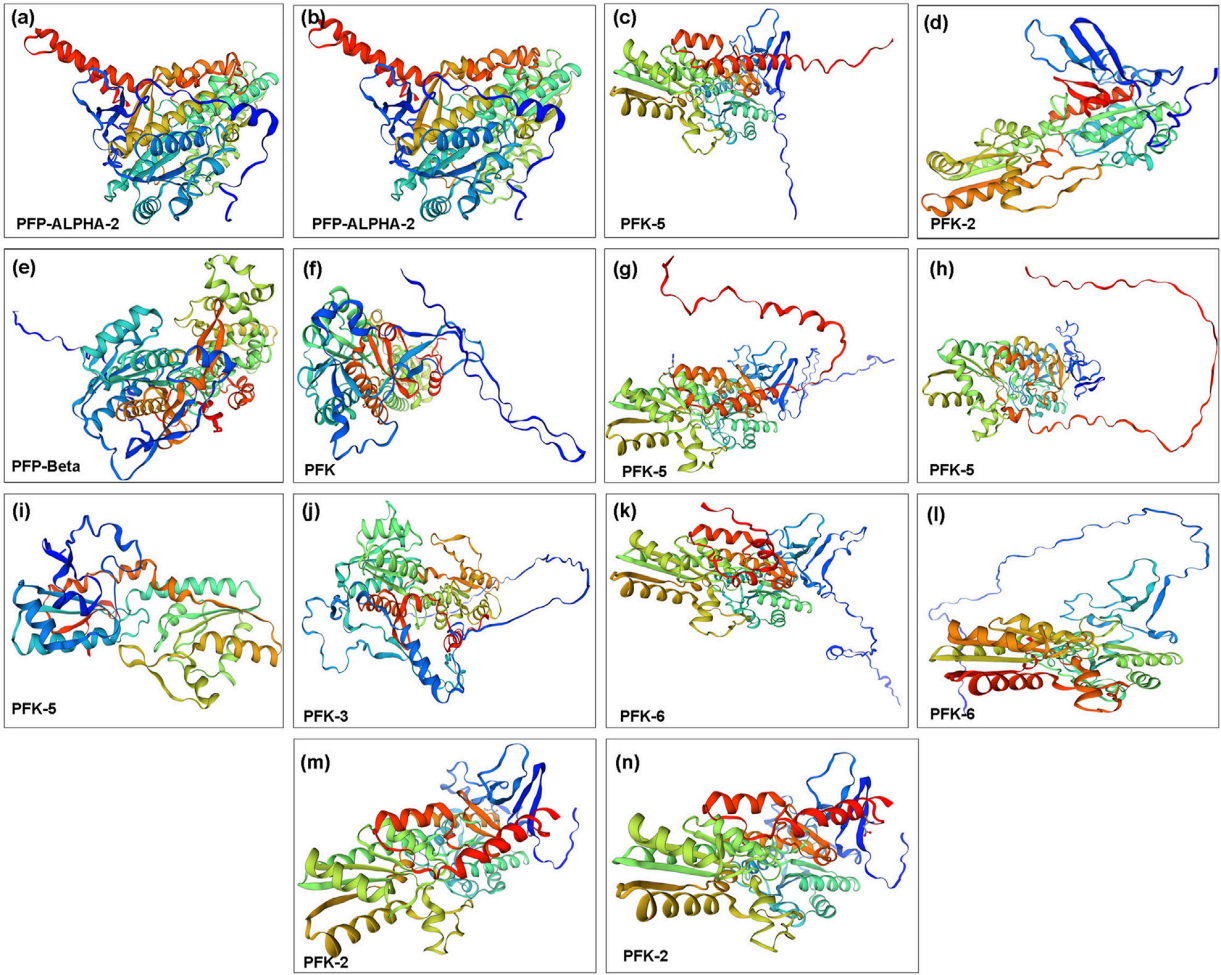
Three-dimensional structure of PFK family proteins. **(A)** Qurub.02G097100.1; **(B)** Qurub.02G097100.2; **(C)** Qurub.02G189400.1; **(D)** Qurub.02G189400.2; **(E)** Qurub.05G067500.1; **(F)** Qurub.05G085700.1; **(G)** Qurub.05G235500.1; **(H)** Qurub.05G235500.2; **(I)** Qurub.05G236300.1; **(J)** Qurub.06G036700.1; **(K)** Qurub.09G134300.1; **(L)** Qurub.09G134300.2; **(M)** Qurub.12G198800.1; **(N)** Qurub.12G198800.2.

The protein’s three-dimensional structure is necessary to understand its functions. All of these proteins contain the same domain architecture, while deducing their structure provides new insights into their function, localization, and interaction network. We used the Swiss-Model server to predict the 3D structure of QrPFK proteins based on the homology modeling approach. Few differences and structural variations were found among the 14 generated protein models. The models with high confidence and identity content were selected. The structural differences of the PFK proteins were consistent with the phylogenetic arrangement. For example, the structures of Qurub.02G097100.1, Qurub.02G097100.2, and Qurub.05G067500.1 proteins were similar and agreed with the results of phylogenetic alignment. These three proteins were identified as PFPs. And the protein structures of the clade containing Qurub.02G189400.1, Qurub.02G189400.2, Qurub.12G198800.1 and Qurub.12G198800.2 also had similar structures. We also found that the members of the clade, Qurub.09G134300.1 and Qurub.09G134300.2, were also similar (Figure 9).

### 3.7 Protein interaction network and KEGG enrichment analysis of QrPFKs

**FIGURE 10:**
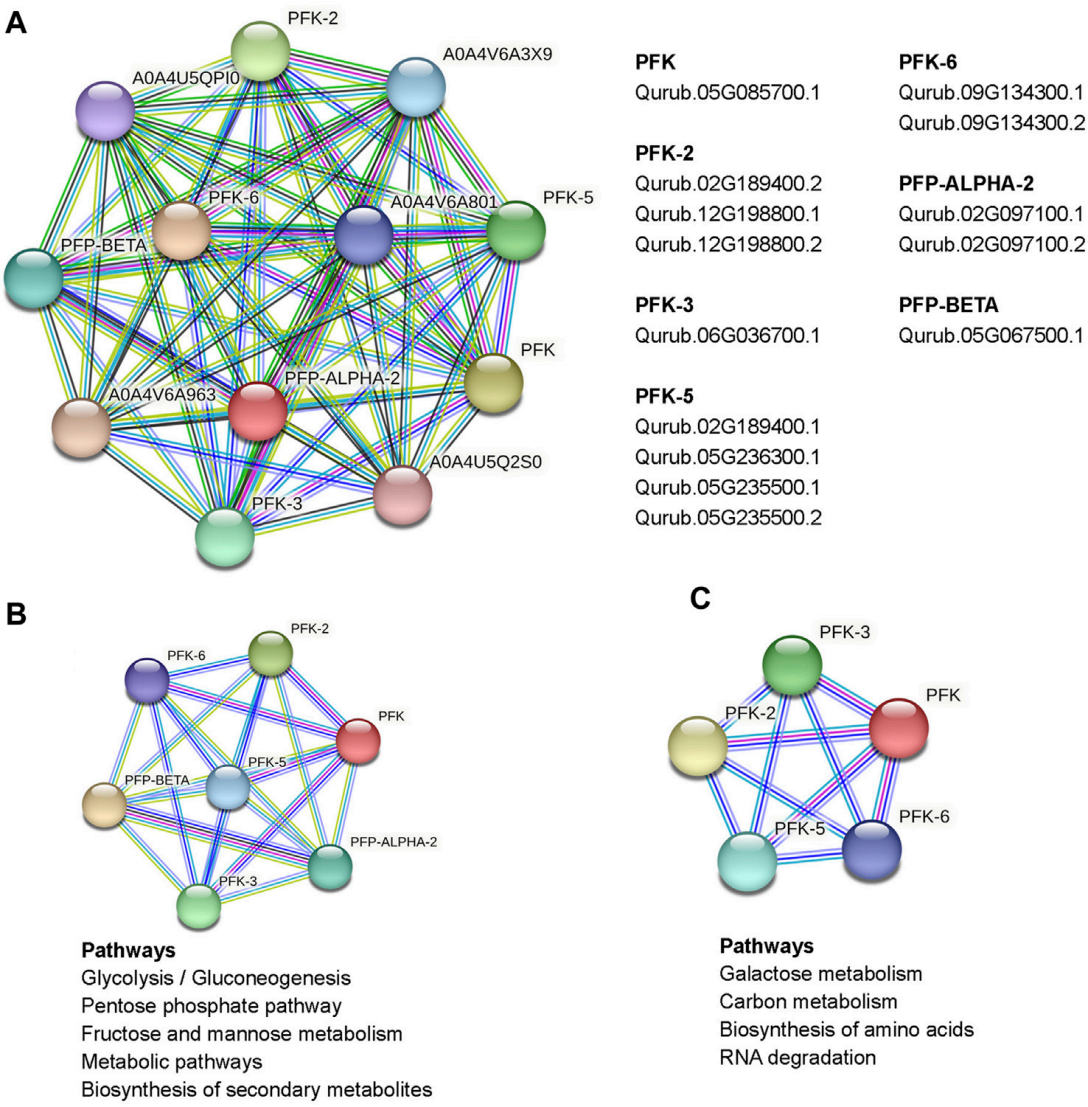
Protein interaction network of PFKs in Quercus rubra, **(A)** emphasized the interact of PFKs with other proteins in *Q. rubra*, **(B)** emphasizes those pathways regulated by interaction between seven PFK family members and **(C)** represents those pathways regulated as a result of interaction of 5 PFK members.

The study of interaction networks can help us better understand the biological processes and molecular pathways involving proteins. Analysis of protein-protein interactions provides important details about the yet unidentified function of a protein. To identify proteins that interact with QrPFKs, we used STRING. A complete network of associations between physical and functional proteins formed the interactions. The results showed that 14 QrPFK proteins interact in different ways with regulatory proteins expressed by few genes unrelated to the PFK gene family (Figure 10). PFK-1 which is shown as PFK, the protein Qurub.05G085700.1 was found to interact with another protein A0A4U5Q2S0 which is known as Phosphotransferase containing the Hexokinase domain. PFK-2 interacts with two proteins from other groups such as Ketose-bisphosphate aldolase class-II family protein (A0A4U5QPI0) and Glucose-6-phosphate isomerase (A0A4V6A3X9). PFP-Beta also interacts with the Ketose-bisphosphate aldolase class-II family protein. Therefore, we observed that PFKs tend to interact with the following proteins such as Phosphotransferase, Ketose-bisphosphate aldolase class-II family protein and Glucose-6-phosphate isomerase. Phosphotransferases are important enzymes in the phosphorylation reaction which mediates the phosphorylation of glucose-6-phosphate. Glucose-6-phosphate isomerase mediates the interconversion of glucose 6-phosphate to fructose-6-phosphate (Figure 10A). Altogether, these enzymes drive the glycolytic process in Q.rubra. KEGG pathway prediction infers that the interaction of seven proteins (PFK, PFK-2, PFK-3, PFK-5, PFK-6, PFP-Alpha2 and PFP-Beta) is responsible for the metabolic pathways, glycolysis/gluconeogenesis, Pentose phosphate pathway, fructose and mannose metabolism and biosynthesis of secondary metabolites (Figure 10B). Also, the interaction between five proteins (PFK, PFK-2, PFK-3, PFK-5 and PFK-6) drives the pathways, galactose metabolism, carbon metabolism, biosynthesis of amino acids and RNA degradation (Figure 10C).

### 3.8 QrPFK expression patterns in different tissues

**FIGURE 11:**
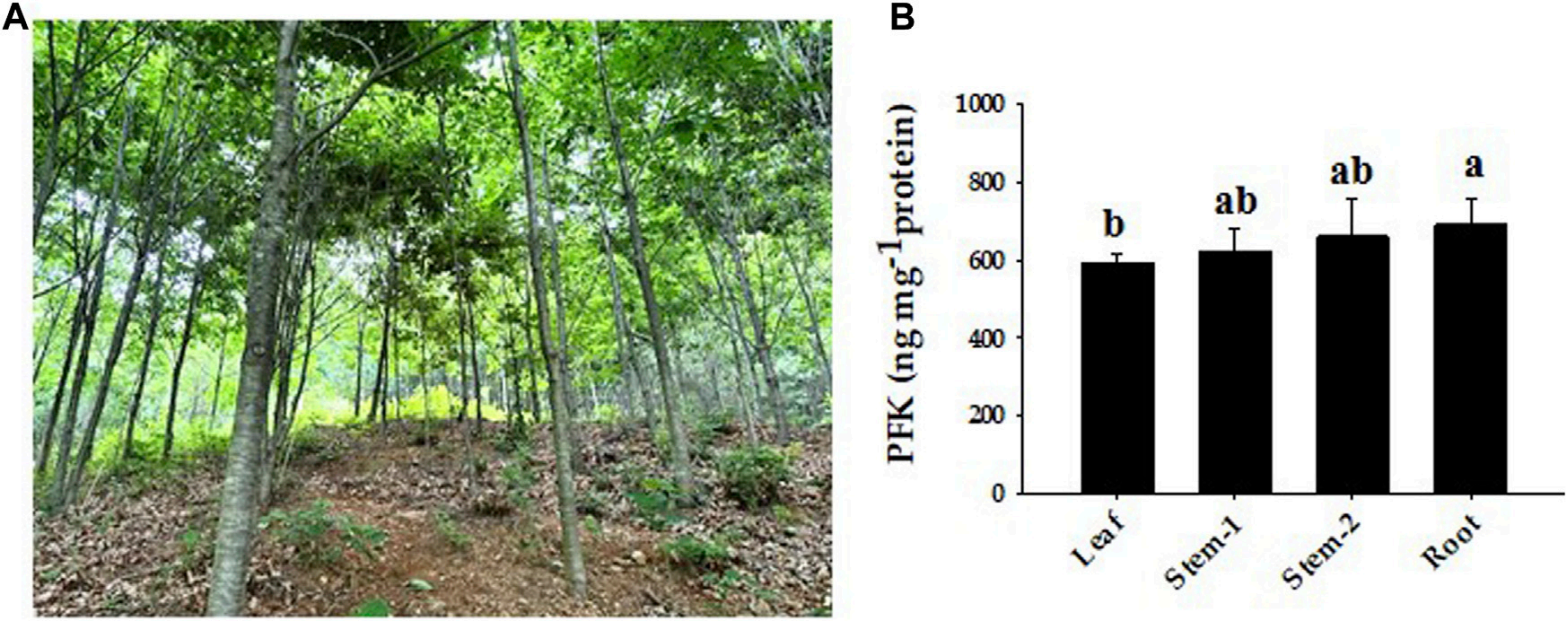
Representative phenotypes of *Quercus rubra* and expression of PFK protein by tissue. **(A)** Growth phenotypes of *Quercus rubra* 7 years after planting. **(B)** PFK concentrations in the leaf, stem, and root of *Quercus rubra*. Stem-1, one-year-old stem (this year’s growth); Stem-2, two-year-old stem (last year’s growth). The results of tests in triplicate are shown as means ± SD. In case of analysis using ANOVA with Tukey’s HSD, significant differences (*p* < 0.05) are indicated by different lowercase letters.

**FIGURE 12:**
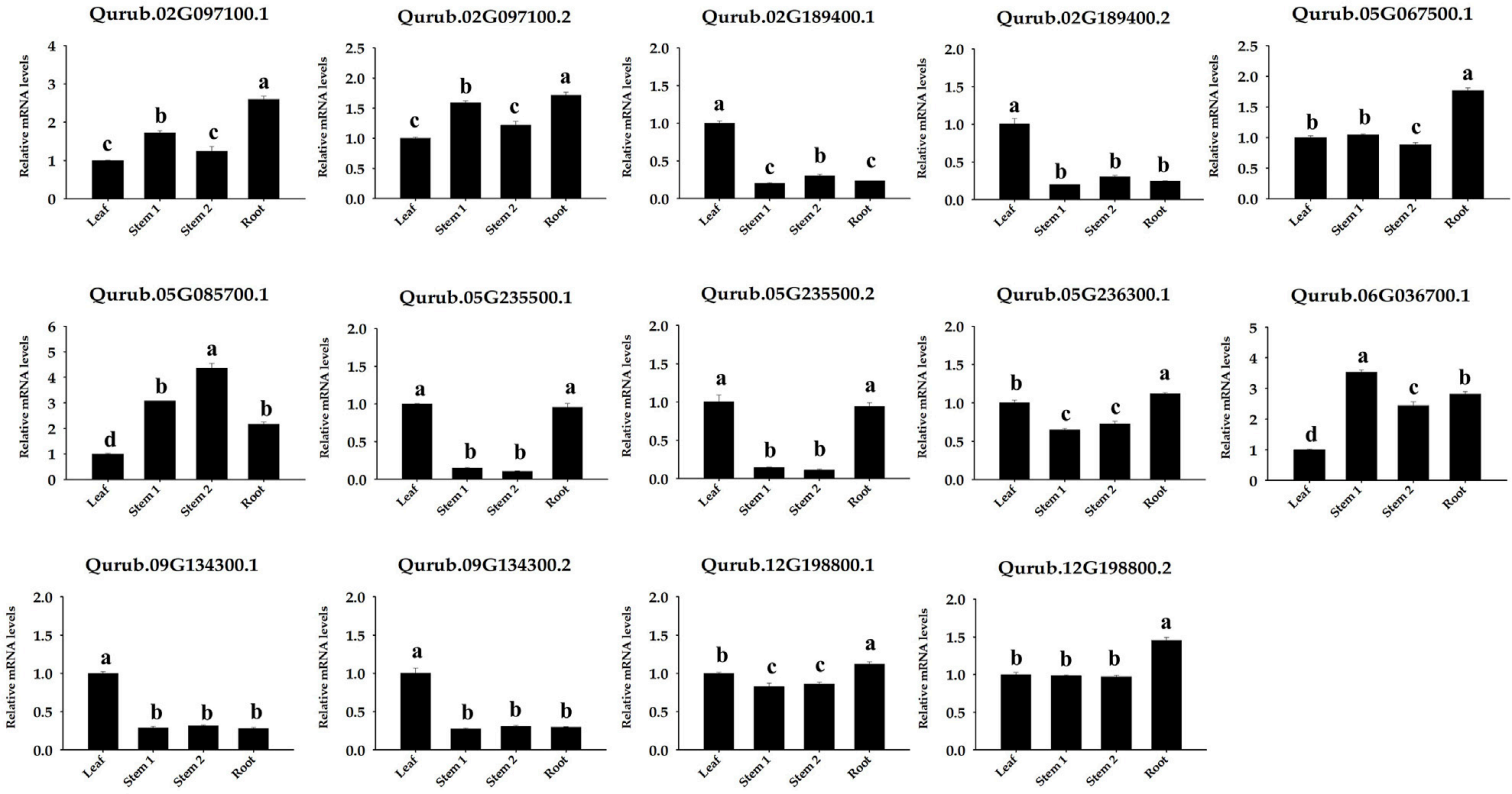
Expression of 14 *PFK* genes in different tissues (leaf, stem, and root) of *Quercus rubra*. Stem-1, one-year-old stem (this year’s growth); Stem-2, two-year-old stem (last year’s growth). Tukey’s test was used to determine whether there were any statistically significant (*p* < 0.05) variations in the levels of *PFK* gene expression. The standard deviation determined from three biological replicates is shown in error bars. Significant differences (*p* < 0.05) are indicated by different lowercase letters.

The amount of PFK was measured in the leaves, stems, and roots to examine the probable function of this enzyme in *Q. rubra* and was confirmed in all tissues (Figure 11). The protein content of *Q. rubra* by tissue was most dominant in the roots. Transcriptional analysis was performed in *Q. rubra* to further examine the expression of *PFP* and *PFK* in various tissues. qPCR was used to evaluate gene expression and determine the roles of *QrPFP* and *QrPFK* in various *Q. rubra* tissues. The expression of all 14 *PFK* genes was observed in different tissues of *Q. rubra*, consistent with the results of the protein assay (Figure 12). Similar to QrPFK, the *QrPFPs* Qurub.02G097100.1, Qurub.02G097100.1, and Qurub.05G067500.1 were expressed in all tissues. In particular, Qurub.05G067500.1 (*QrPFPβ*) was highly expressed in the roots (Figure 12). QrPFP may play a critical role in the glycolytic pathway of *Q. rubra* when it coexists with QrPFK. Additionally, tissue-specific expression patterns of the *PFK* genes were identified. Qurub.02G189400.1 (*QrPFK*), Qurub.02G189400.2 (*QrPFK*), Qurub.09G134300.1 (*QrPFK*), and Qurub.09G134300.2 (*QrPFK*) were specifically expressed in leaves, Qurub.05G085700.1 (*QrPFK*) in stem tissue, and Qurub.05G067500.1 (*QrPFPβ*) in roots. Furthermore, two *QrPFK* genes, Qurub.05G235500.1 and Qurub.05G235500.2, were expressed highly in leaves and roots and weakly in stems (Figure 12). We examined the correlation between protein levels in tissues and gene expression levels in qPCR. Qurub.02G097100.1 and Qurub.12G198800.2 showed a strong positive correlation, and Qurub.02G189400.1, Qurub.02G189400.2, Qurub.09G134300.1 and Qurub.09G134300.2 showed a strong negative correlation. Additionally, a clear positive correlation was confirmed between Qurub.02G097100.2 and Qurub.05G067500.1. However, Qurub.05G085700.1, Qurub.05G235500.1, Qurub.05G235500.2, Qurub.05G236300.1, Qurub.06G036700.1, and Qurub.12G198800.1 did not show a statistically significant linear correlation with protein levels (Supplementary Table S3). These genes could be used to exploit tissue-specific expression patterns. These results provide a theoretical foundation for further exploration of the molecular characteristics and biological functions of QrPFK and QrPFP.

## 4 Discussion

Red oak (*Q. rubra* L.) is an intermediate shade-tolerant tree species that, in the absence of disturbances (i.e., fire), is succeeded by more shade-tolerant competitors (Chhin, 2018). The *PFK* gene family plays a critical role in plant growth and development, particularly during the early phases. This gene family is well known for its highly conserved 6-PFK domain, which is responsible for the glycolytic activity of PFK proteins. This study provides extensive data on 14 *QrPFK* sequences. We also compared the population of the PFK gene family in other crops from previous studies. The number of *PFK* genes in *Quercus* (*n* = 14) was much higher than that in *Arabidopsis* (*n* = 12), potato (*n* = 13), and castor bean (*n* = 9) but lower than that in *Populus* (*n* = 17) and rice (*n* = 15). In addition, the presence of PFKs were also recorded in several other species such as *Fragaria vesca, Prunus mume, Prunus persica, Pyrus x bretschneideri and Malus domestica* (Lü et al., 2019). We also confirmed their localization in *Q*. *rubra* chromosomes like other studies. The potential evolutionary processes of *Q. rubra* were inferred at both interspecific and intraspecific levels. Chromosomal mapping of the 14 *QrPFK* genes indicated that they were unevenly distributed along the 12 linkage groups/chromosomes. Almost five genes (four from *QrPFK* and one from *QrPFP*) were located on chromosome LG05, whereas four genes (two from *QrPFK* and two from *QrPFP*) were located on chromosome LG02. In LG06, only one gene was detected (Figure 6). Physicochemical studies are more important to understand the nature of any proteins mainly focusing on the molecular weight, theoretical pI, aliphatic index, and GRAVY (Wang et al., 2021). Several research supports our prediction that PFKs are hydrophilic in nature. These hydrophilic proteins due to their polar nature can readily form hydrogen bonds with water molecules and aliphatic index has predicted that PFKs are stable proteins even at higher temperature. These properties are satisfying because previous studies suggest that these proteins have been involved in most of the biological processes such as cell signaling, transport of molecules across cell membranes and enzymatic reactions. Hydrophilic proteins mostly safeguard the cells against stress response (Guo et al., 2021). Phosphofructokinase, is one of the vital rate-determining enzymes for cellular respiration, like pyruvate dehydrogenase and isocitrate dehydrogenase (Megguer et al., 2017). Under normal circumstances, plants utilize PFK-2 for their cellular respiration but when plants undergo oxidative stress and consequently at low ATP levels, plants utilize the alternative PFP based cellular respiration (Megguer et al., 2017). First, the sequences of all PFK members were aligned to examine for visible differences among them. We observed that three proteins, Qurub.05G067500.1, Qurub.02G097100.1, and Qurub.02G097100.2, showed numerous deletions compared to the other QrPFK proteins (Figure 3) and found to be less conserved. However, the sequences appeared conserved between *QrPFK* members of PFK superfamily. Phylogenetic analysis with the PFK gene family members with other reported crops such as *A*. *thaliana, P. deltoides, R. communis*, *O. sativa*, and *S. tuberosum* has grouped Qurub.05G067500.1, Qurub.02G097100.1, and Qurub.02G097100.2 with the PFPs of other model crops. Similar instances were observed in the crops, *P. deltoides* (Kim et al., 2023)*, A. thaliana* (Mustroph et al., 2007), *R. communis* (Podesta and Plaxton, 1994), *O. sativa* (Kato-Noguchi, 2002), and *S. tuberosum* (Kim et al., 2023), implying that they belonged to PFP family.

The availability of the *Q. rubra* genome facilitated the investigation of the roles of PFKs and PFPs. Generally, *PFK* family genes are intron-rich, with more than 12 introns. *PFP* genes were found to be more intron-rich than *PFK* members during the analysis of the exon-intron structure (Figure 5). Most eukaryotes are intron-rich; in particular, the higher the intron population, the higher the chance of alternative splicing into multiple transcripts. This process leads to the formation of different proteins and plays a pivotal role in adaptation and evolution (Liu et al., 2021). Similar conditions have been observed in cassava with an intron count >15 (Wang et al., 2021). All three *QrPFP* members possess conserved intron-exon structures. In addition, motif analysis showed that PFP proteins lost most of their motifs, such as 3, 4, and 6, because motifs 1–6, except 5, are significant in determining the glycolytic activity of PFK family members. They act as proton acceptor and allows binding of ATP on PFK. Among the detected motifs, the function of motif 5 is not yet predicted which might involve in the pyrophosphate-dependent phosphofructokinase reaction. We observed that motifs 1 and 2 only supported glycolytic motifs in the PFP members. In cotton, PFPs hold only two to four motifs responsible for glycolytic activity in PFK, indicating that universally, PFPs have limited motifs due to large deletions in their amino acid sequences (Mehari et al., 2022).

The asymmetrical arrangement of genes may provide information regarding their evolution (Chen et al., 2016). WGD or polyploidization may be the main driving force behind the evolution of novel characteristics and new transcriptional regulatory sites that can change expression patterns (Panchy et al., 2016). Three homologous pairings were verified because of the tandem duplication. Tandem duplication also plays an adaptive role in the growth and function of abiotic stress-responsive genes. Tandem repetitions frequently share identical *cis-*acting components (Flagel and Wendel, 2009). Our analysis also highlights the possibility that tandem gene duplication pairs in *QrPFKs* may have comparable roles and regulatory components in their promoter regions. In *Arabidopsis*, the expression of 15 phosphofructokinase-encoding genes were studied in roots and aerial tissues of anoxia-tolerant seedlings in response to anoxic stress (Mustroph et al., 2013). Similarly, in anoxic stress, 15 genes encoding PFKs were reported (Kato-Noguchi, 2002) and earlier studies have reported the presence of PFK genes in castor and potato. Therefore, we studied synteny with other species, including *P. deltoides*, *A. thaliana*, *O. sativa*, *R. communis*, and *S. tuberosum*, revealed to be syntenic (Figure 7A). Collinearity analysis revealed three intraspecific gene pairs (Qurub.12G198800.1. v2.1/Qurub.02G189400.1.v2.1, Qurub.12G198800.1.v2.1/Qurub.05G235500.1. v2.1 and Qurub.02G189400.1.v2.1/Qurub.05G235500.1.v2.1) were segmentally duplicated. The Ka/Ks values of the collinear pairs were studied to understand the drivers of gene evolution. Almost all three pairs were suspected to have undergone simultaneous duplication.

Genome-wide analysis revealed differences in the number of CREs across the 14 *QrPFK* genes. CREs, such as the ABA-responsive element (ABRE), are important components of the abscisic acid pathway and are involved in ABA-mediated oxidative stress (Wang et al., 2021). Compared to the TATA box and CAAT box, the levels of stress-related CREs were very low. A slight increase in their levels compared to other CREs indicates that these genes are involved in the stress experienced by trees during development. The TATA box-binding protein, a crucial component in identifying core promoter regions, recognizes the TATA box (Haberle and Stark, 2018; Vo Ngoc et al., 2019). However, most fundamental promoters in plants lack TATA, and additional *cis-*elements are involved in the initiation of transcription at these promoters. Similary, these *cis*-elements were also reported in B3 gene family in soybean (Ren et al., 2023), MDH gene family in rice (Zhang et al., 2022) and PFK gene family in cotton (Mehari et al., 2022).

Analysis of QrPFKs tertiary structure and protein-protein interaction is helpful to better understand the function of QrPFKs genes. In this study, genes in the same branch have similar protein structures, such as Qurub.02G097100.1, Qurub.02G097100.2, and Qurub.05G067500.1, so they may have similar functions. Similarly, Qurub.02G189400.1, Qurub.02G189400.2, Qurub.12G198800.1 and Qurub.12G198800.2 may have similar functions. Similar structures in Qurub.02G189400.1, Qurub.02G189400.2, Qurub.12G198800.1 and Qurub.12G198800.2 also confer the same functions. The constructed three-dimensional structures were validated using Ramachandran plot. In all these structures, more than 92% of the residues were in the most allowed regions which confirms that the predicted models were anticipated as natural protein structures (Supplementary Figure S3). In addition, we constructed a network of protein interactions in *Populus* orthologs of *Quercus*. We found that the proteins phosphotransferase, which contains the hexokinase domain, ketose-bisphosphate aldolase class protein II, and glucose-6-phosphate isomerase belong to the glycolysis pathway responsible for the conversion of glucose to pyruvate. The ketose-bisphosphate aldolase class- II family protein is classified as fructose-1,6-bisphosphate aldolase (Zhao et al., 2019). Numerous studies have demonstrated the importance of PFP activity in plants. For example, mutant rice plants have produced grains that resemble flour and have lower endosperm thickness and total starch content without affecting vegetative or reproductive development of the plants. (Duan et al., 2016).

The expression patterns of these three pairs of genes were similar in leaves. In addition, the expression of Qurub.02G189400.1. v2.1 was lower than that of Qurub.12G198800.1.v2.1 and Qurub.05G235500.1.v2.1 in stems and roots. We observed that *QrPFKs* and *QrPFPs* were expressed in all tissues, including the leaves, stems, and roots. *Quercus* has only two *PFPα* genes and only one *PFPβ*. A previous study reported that the well-known *PFPβ* gene of *A. thaliana* showed a different expr ession pattern from the dominant expression in the leaf (Lim et al., 2009). However, *QrPFPβ* expression was higher in the roots than in other tissues. These findings imply that the role of the PFPβ gene in *Quercus* may be different from that in *Arabidopsis*.

## 5 Conclusion

In plants, PFP performs various functions during glycolysis by reversibly converting fructose-6-phosphate and pyrophosphate into fructose-1,6-bisphosphate and orthophosphate. PFP belongs to the phosphofructokinase gene family. In the present study, we identified 11 *QrPFK* and 3 *QrPFP* family members based on the latest genome of *Q. rubra*. The *PFK* gene consists of 12 introns or less, and the *PFP* gene consists of 15 or more introns. In addition, these 14 genes were distributed in a biased manner on five chromosomes. Two motifs (1 and 2) were observed to be conserved in these 14 proteins. Subcellular localization analysis revealed that they primarily existed in the cytoplasm, chloroplasts, and cytoskeleton. Phytohormone response, plant growth and development, and stress-related CREs have been found to be most prevalent and relevant in the *QrPFK* promoter. Tissue-specific proteins were expressed in leaves, stems, and roots and were particularly high in roots. Expression profile analysis using qPCR revealed that *PFPβ* was highly expressed in the roots, irrespective of its behavior in other plant species. This study provides a foundation for further research on the molecular mechanisms of PFK proteins in the development of *Q. rubra*.

## Data Availability

The original contributions presented in the study are included in the article/Supplementary Material, further inquiries can be directed to the corresponding author.

## References

[B1] Bapteste E. Moreira D. Philippe H. (2003). Rampant horizontal gene transfer and phospho-donor change in the evolution of the phosphofructokinase. Gene 318, 185–191. 10.1016/s0378-1119(03)00797-2 14585511

[B2] Cawood M. E. Botha F. C. Small J. C. (1988). Properties of the phosphofructokinase isoenzymes from germinating cucumber seeds. J. Plant Physiol. 132 (2), 204–209. 10.1016/s0176-1617(88)80162-7

[B3] Chen S.-C. Cannon C. H. Kua C.-S. Liu J.-J. Galbraith D. W. (2014). Genome size variation in the *Fagaceae* and its implications for trees. Tree Genet. Genomes 10, 977–988. 10.1007/s11295-014-0736-y

[B4] Chen L. Han J. Deng X. Tan S. Li L. Li L. (2016). Expansion and stress responses of AP2/EREBP superfamily in *Brachypodium distachyon* . Sci. Rep. 6 (1), 21623. 10.1038/srep21623 26869021 PMC4751504

[B5] Chen C. Chen H. Zhang Y. Thomas H. R. Frank M. H. He Y. (2020). TBtools: an integrative toolkit developed for interactive analyses of big biological data. Mole. Plant 13 (8), 1194–1202. 10.1016/j.molp.2020.06.009 32585190

[B6] Chhin S. (2018). Managing red oak (*Quercus rubra* L.) reduces sensitivity to climatic stress. J. For. Environ. Sci. 34 (4), 338–351. 10.7747/JFES.2018.34.4.338

[B7] Cui X. Fan B. Scholz J. Chen Z. (2007). Roles of *Arabidopsis* cyclin-dependent kinase C complexes in cauliflower mosaic virus infection, plant growth, and development. Plant Cell 19 (4), 1388–1402. 10.1105/tpc.107.051375 17468259 PMC1913762

[B8] Darikova Y. A. Sherbakov D. Y. (2009). Evolution of a phosphofructokinase gene intron in gastropods of the family *Baicaliidae* . Mol. Biol. 43, 776–782. 10.1134/s0026893309050094 19899631

[B9] Duan E. Wang Y. Liu L. Zhu J. Zhong M. Zhang H. (2016). Pyrophosphate: fructose-6-phosphate 1-phosphotransferase (PFP) regulates carbon metabolism during grain filling in rice. Plant Cell Rep. 35, 1321–1331. 10.1007/s00299-016-1964-4 26993329 PMC4869756

[B10] Dunaway G. A. (1983). A review of animal phosphofructokinase isozymes with an emphasis on their physiological role. Mole. Cell. Biochem. 52, 75–91. 10.1007/BF00230589 6306441

[B11] Eddy S. R. (2009). “A new generation of homology search tools based on probabilistic inference,” in Genome informatics 2009: genome informatics series (World Scientific), 23, 205–211. 20180275

[B12] Flagel L. E. Wendel J. F. (2009). Gene duplication and evolutionary novelty in plants. New Phytol. 183 (3), 557–564. 10.1111/j.1469-8137.2009.02923.x 19555435

[B13] Godfrey R. K. (1988). Trees, shrubs, and woody vines of northern Florida and adjacent Georgia and Alabama. University of Georgia Press.

[B53] Goodstein D. M. Shu S. Howson R. Neupane R. Hayes R. D. Fazo J. (2012). Phytozome: a comparative platform for green plant genomics. Nucleic acids Res. 40 (D1), D1178–D1186. 10.1093/nar/gkr944 22110026 PMC3245001

[B14] Guo L. Zhao M. Tang Y. Han J. Gui Y. Ge J. (2021). Modular assembly of ordered hydrophilic proteins improve salinity tolerance in *Escherichia coli* . Int. J. Mol. Sci. 22 (9), 4482. 10.3390/ijms22094482 33923104 PMC8123400

[B15] Haberle V. Stark A. (2018). Eukaryotic core promoters and the functional basis of transcription initiation. Nat. Rev. Mol. Cell Biol. 19 (10), 621–637. 10.1038/s41580-018-0028-8 29946135 PMC6205604

[B16] Horton P. Park K.-J. Obayashi T. Fujita N. Harada H. Adams-Collier C. (2007). WoLF PSORT: protein localization predictor. Nucleic Acids Res. 35 (2), W585–W587. 10.1093/nar/gkm259 17517783 PMC1933216

[B17] Hu B. Jin J. Guo A.-Y. Zhang H. Luo J. Gao G. (2015). GSDS 2.0: an upgraded gene feature visualization server. Bioinformatics 31 (8), 1296–1297. 10.1093/bioinformatics/btu817 25504850 PMC4393523

[B18] Hurst L. D. (2002). The Ka/Ks ratio: diagnosing the form of sequence evolution. Trends Genet. 18 (9), 486–487. 10.1016/s0168-9525(02)02722-1 12175810

[B19] Initiative I. P. G. Verde I. Abbott A. G. Scalabrin S. Jung S. Shu S. (2013). The high-quality draft genome of peach (*Prunus persica*) identifies unique patterns of genetic diversity, domestication and genome evolution. Nat. Genet. 45 (5), 487–494. 10.1038/ng.2586 23525075

[B20] Isaac J. Rhodes M. (1982). Purification and properties of phosphofructokinase from fruits of *Lycopersicon esculentum* . Phytochemistry 21 (7), 1553–1556. 10.1016/s0031-9422(82)85016-4

[B21] Jiao Y. Wickett N. J. Ayyampalayam S. Chanderbali A. S. Landherr L. Ralph P. E. (2011). Ancestral polyploidy in seed plants and angiosperms. Nature 473 (7345), 97–100. 10.1038/nature09916 21478875

[B22] Kato‐Noguchi H. (2002). The catalytic direction of pyrophosphate: fructose 6‐phosphate 1‐phosphotransferase in rice coleoptiles in anoxia. Physiol. Plant. 116 (3), 345–350. 10.1046/j.0031-9317.2002.00002.x

[B23] Kim T.-L. Denison M. I. J. Lim H. Chung H. Oh C. (2023). Genome-wide analysis, identification, and characterization of the *PFK* gene family members of *Populus deltoides* . Forests 14 (6), 1104. 10.3390/f14061104

[B24] Knowles V. L. Greyson M. F. Dennis D. T. (1990). Characterization of ATP-dependent fructose 6-phosphate 1-phosphotransferase isozymes from leaf and endosperm tissues of *Ricinus communis* . Plant Physiol. 92 (1), 155–159. 10.1104/pp.92.1.155 16667239 PMC1062263

[B25] Kremer A. Casasoli M. Barreneche T. Bodénès C. Sisco P. Kubisiak T. (2007). “Fagaceae trees,” in Forest trees, 161–187.

[B26] Lescot M. Déhais P. Thijs G. Marchal K. Moreau Y. Van de Peer Y. (2002). PlantCARE, a database of plant *cis*-acting regulatory elements and a portal to tools for *in silico* analysis of promoter sequences. Nucleic Acids Res. 30 (1), 325–327. 10.1093/nar/30.1.325 11752327 PMC99092

[B27] Letunic I. Bork P. (2007). Interactive Tree of Life (iTOL): an online tool for phylogenetic tree display and annotation. Bioinformatics 23 (1), 127–128. 10.1093/bioinformatics/btl529 17050570

[B28] Li J.-m. Zheng D.-m. Li L.-t. Qiao X. Wei S.-w. Bai B. (2015). Genome-wide function, evolutionary characterization and expression analysis of sugar transporter family genes in pear (*Pyrus bretschneideri* Rehd). Plant Cell Physiol. 56 (9), 1721–1737. 10.1093/pcp/pcv090 26079674

[B29] Li J. Qin M. Qiao X. Cheng Y. Li X. Zhang H. (2017). A new insight into the evolution and functional divergence of SWEET transporters in Chinese white pear (*Pyrus bretschneideri*). Plant Cell Physiol. 58 (4), 839–850. 10.1093/pcp/pcx025 28339862

[B30] Lim H. Cho M.-H. Jeon J.-S. Bhoo S. H. Kwon Y.-K. Hahn T.-R. (2009). Altered expression of pyrophosphate: fructose-6-phosphate 1-phosphotransferase affects the growth of transgenic *Arabidopsis* plants. Mol. Cells 27, 641–649. 10.1007/s10059-009-0085-0 19533038

[B31] Lim H. Cho M.-H. Bhoo S. H. Hahn T.-R. (2014). Pyrophosphate: fructose-6-phosphate 1-phosphotransferase is involved in the tolerance of *Arabidopsis* seedlings to salt and osmotic stresses. Vitro Cel. Dev. Biol. Plant 50, 84–91. 10.1007/s11627-013-9578-9

[B32] Liu S. Liu Y. Yang X. Tong C. Edwards D. Parkin I. A. (2014). The *Brassica oleracea* genome reveals the asymmetrical evolution of polyploid genomes. Nat. Commun. 5 (1), 3930. 10.1038/ncomms4930 24852848 PMC4279128

[B33] Liu H. Lyu H. M. Zhu K. Van de Peer Y. Cheng Z. M. (2021). The emergence and evolution of intron‐poor and intronless genes in intron‐rich plant gene families. Plant J. 105 (4), 1072–1082. 10.1111/tpj.15088 33217085 PMC7116809

[B34] Livak K. J. Schmittgen T. D. (2001). Analysis of relative gene expression data using real-time quantitative PCR and the 2^− ΔΔ^CT method. Methods 25 (4), 402–408. 10.1006/meth.2001.1262 11846609

[B35] Lü H. Li J. Huang Y. Zhang M. Zhang S. Wu J. (2019). Genome-wide identification, expression and functional analysis of the phosphofructokinase gene family in Chinese white pear (*Pyrus bretschneideri*). Gene 702, 133–142. 10.1016/j.gene.2019.03.005 30904717

[B36] Mahajan R. Singh R. (1992). Properties of ATP-dependent phosphofructokinase from endosperm of developing wheat (*Triticum aestivum L.*) grains. J. Plant Biochem. Biotechnol. 1, 45–48. 10.1007/bf03262894

[B37] Makela M. Michael P. Theriault G. Nkongolo K. (2016). High genetic variation among closely related red oak (*Quercus rubra*) populations in an ecosystem under metal stress: analysis of gene regulation. Genes Genomics 38, 967–976. 10.1007/s13258-016-0441-3

[B38] Megguer C. A. Fugate K. K. Lafta A. M. Ferrareze J. P. Deckard E. L. Campbell L. G. (2017). Glycolysis is dynamic and relates closely to respiration rate in stored sugarbeet roots. Front. Plant Sci. 8, 861. 10.3389/fpls.2017.00861 28596778 PMC5442176

[B39] Mehari T. G. Xu Y. Umer M. J. Hui F. Cai X. Zhou Z. (2022). Genome-wide identification and expression analysis elucidates the potential role of *PFK* gene family in drought stress tolerance and sugar metabolism in cotton. Front. Genet. 13, 922024. 10.3389/fgene.2022.922024 35795210 PMC9251378

[B40] Migueal P. Nkongolo K. Michael P. Djeukam C. (2017). Differential gene transcription in red oak (*Quercus rubra*) genotypes resistant to copper toxicity. Am. J. Biochem. Biotechnol. 13 (4), 215–225. 10.3844/ajbbsp.2017.215.225

[B41] Mustroph A. Sonnewald U. Biemelt S. (2007). Characterisation of the ATP-dependent phosphofructokinase gene family from *Arabidopsis thaliana* . FEBS Lett. 581 (13), 2401–2410. 10.1016/j.febslet.2007.04.060 17485088

[B42] Mustroph A. Stock J. Hess N. Aldous S. Dreilich A. Grimm B. (2013). Characterization of the phosphofructokinase gene family in rice and its expression under oxygen deficiency stress. Front. Plant Sci. 4, 125. 10.3389/fpls.2013.00125 23717315 PMC3653104

[B43] Ohno S. (1970). The enormous diversity in genome sizes of fish as a reflection of natureˈs extensive experiments with gene duplication. Trans. Am. Fish. Soc. 99 (1), 120–130. 10.1577/1548-8659(1970)99<120:tedigs>2.0.co;2

[B44] Panchy N. Lehti-Shiu M. Shiu S.-H. (2016). Evolution of gene duplication in plants. Plant physiol. 171 (4), 2294–2316. 10.1104/pp.16.00523 27288366 PMC4972278

[B45] Panozzo A. Dal Cortivo C. Ferrari M. Vicelli B. Varotto S. Vamerali T. (2019). Morphological changes and expressions of AOX1A, CYP81D8, and putative PFP genes in a large set of commercial maize hybrids under extreme waterlogging. Front. Plant Sci. 10, 62. 10.3389/fpls.2019.00062 30778365 PMC6369177

[B46] Plaxton W. C. (1996). The organization and regulation of plant glycolysis. Annu. Rev. Plant Biol. 47 (1), 185–214. 10.1146/annurev.arplant.47.1.185 15012287

[B47] Podestá F. E. Plaxton W. C. (1994). Regulation of cytosolic carbon metabolism in germinating *Ricinus communis* cotyledons: I. Developmental profiles for the activity, concentration, and molecular structure of the pyrophosphate-and ATP-dependent phosphofructokinases, phospho enol pyruvate carboxylase and pyruvate kinase. Planta 194 (3), 374–380. 10.1007/bf00197538

[B48] Qiao X. Li M. Li L. Yin H. Wu J. Zhang S. (2015). Genome-wide identification and comparative analysis of the heat shock transcription factor family in Chinese white pear (*Pyrus bretschneideri*) and five other Rosaceae species. BMC Plant Biol. 15 (1), 12–16. 10.1186/s12870-014-0401-5 25604453 PMC4310194

[B49] Qin Q. Kaas Q. Wu W. Lin F. Lai Q. Zhu Z. (2014). Characterisation of the subunit genes of pyrophosphate-dependent phosphofructokinase from loquat (*Eriobotrya japonica* Lindl.). Tree Genet. Genomes 10, 1465–1476. 10.1007/s11295-014-0774-5

[B51] Ren C. Wang H. Zhou Z. Jia J. Zhang Q. Liang C. (2023). Genome-wide identification of the B3 gene family in soybean and the response to melatonin under cold stress. Front. Plant Sci. 13, 1091907. 10.3389/fpls.2022.1091907 36714689 PMC9880549

[B52] Sander I. L. (1990). *Quercus rubra* L. Northern red oak. Silvics N. Am. 2, 727–733.

[B54] Shulaev V. Sargent D. J. Crowhurst R. N. Mockler T. C. Folkerts O. Delcher A. L. (2011). The genome of woodland strawberry (*Fragaria vesca*). Nat. Genet. 43 (2), 109–116. 10.1038/ng.740 21186353 PMC3326587

[B55] Siebers B. Klenk H.-P. Hensel R. (1998). PPi-dependent phosphofructokinase from Thermoproteus tenax, an archaeal descendant of an ancient line in phosphofructokinase evolution. J. Bacteriol. 180 (8), 2137–2143. 10.1128/JB.180.8.2137-2143.1998 9555897 PMC107141

[B56] Sievers F. Higgins D. G. (2014). Clustal omega. Curr. Protoc. Bioinforma. 48 (1), 1.16. 11–13.13. 16. 10.1002/0471250953.bi0313s48 25501942

[B57] Straigyte L. Zalkauskas R. (2012). Effect of climate variability on *Quercus rubra* phenotype and spread in Lithuanian forests. Dendrobiology 67.

[B58] Szklarczyk D. Gable A. L. Lyon D. Junge A. Wyder S. Huerta-Cepas J. (2019). STRING v11: protein–protein association networks with increased coverage, supporting functional discovery in genome-wide experimental datasets. Nucleic Acids Res. 47 (D1), D607–D613. 10.1093/nar/gky1131 30476243 PMC6323986

[B59] Teramoto M. Koshiishi C. Ashihara H. (2000). Wound-induced respiration and pyrophosphate: fructose-6-phosphate phosphotransferase in potato tubers. Z. Naturforsch. C 55 (11-12), 953–956. 10.1515/znc-2000-11-1217 11204201

[B60] Thompson J. D. Gibson T. J. Higgins D. G. (2003). Multiple sequence alignment using ClustalW and ClustalX. Curr. Protoc. Bioinforma. 00 (1), 2–3. 10.1002/0471250953.bi0203s00 18792934

[B61] Turner W. L. Plaxton W. C. (2003). Purification and characterization of pyrophosphate-and ATP-dependent phosphofructokinases from banana fruit. Planta 217, 113–121. 10.1007/s00425-002-0962-7 12721855

[B62] Velasco R. Zharkikh A. Affourtit J. Dhingra A. Cestaro A. Kalyanaraman A. (2010). The genome of the domesticated apple (*Malus*× *domestica* Borkh.). Nat. Genet. 42 (10), 833–839. 10.1038/ng.654 20802477

[B63] Vo Ngoc L. Kassavetis G. A. Kadonaga J. T. (2019). The RNA polymerase II core promoter in *Drosophila* . Genetics 212 (1), 13–24. 10.1534/genetics.119.302021 31053615 PMC6499525

[B64] Wang H. Zhao P. Shen X. Xia Z. Zhou X. Chen X. (2021). Genome-wide survey of the phosphofructokinase family in cassava and functional characterization in response to oxygen-deficient stress. BMC Plant Biol. 21, 376–415. 10.1186/s12870-021-03139-7 34399701 PMC8365977

[B65] Wegener G. Krause U. (2002). Different modes of activating phosphofructokinase, a key regulatory enzyme of glycolysis, in working vertebrate muscle. Biochem. Soc. Trans. 30 (2), 264–270. 10.1042/bst0300264 12023862

[B66] Winkler C. Delvos B. Martin W. Henze K. (2007). Purification, microsequencing and cloning of spinach ATP‐dependent phosphofructokinase link sequence and function for the plant enzyme. FEBS J. 274 (2), 429–438. 10.1111/j.1742-4658.2006.05590.x 17229148

[B67] Wu J. Wang Z. Shi Z. Zhang S. Ming R. Zhu S. (2013). The genome of the pear (*Pyrus bretschneideri* Rehd.). Genome Res. 23 (2), 396–408. 10.1101/gr.144311.112 23149293 PMC3561880

[B68] Yang Z. Nielsen R. (2000). Estimating synonymous and nonsynonymous substitution rates under realistic evolutionary models. Mol. Biol. Evol. 17 (1), 32–43. 10.1093/oxfordjournals.molbev.a026236 10666704

[B69] Zhang Q. Chen W. Sun L. Zhao F. Huang B. Yang W. (2012). The genome of *Prunus mume* . Nat. Commun. 3 (1), 1318. 10.1038/ncomms2290 23271652 PMC3535359

[B70] Zhang Y. Wang Y. Sun X. Yuan J. Zhao Z. Gao J. (2022). Genome-wide identification of MDH family genes and their association with salt tolerance in rice. Plants 11 (11), 1498. 10.3390/plants11111498 35684271 PMC9182821

[B71] Zhang J. (2003). Evolution by gene duplication: an update. Trends Ecol. Evol. 18 (6), 292–298. 10.1016/s0169-5347(03)00033-8

[B72] Zhao W. Liu H. Zhang L. Hu Z. Liu J. Hua W. (2019). Genome-wide identification and characterization of FBA gene family in polyploid crop *Brassica napus* . Int. J. Mol. Sci. 20 (22), 5749. 10.3390/ijms20225749 31731804 PMC6888112

[B73] Zhu C. Chen Z. Yu G. (2013). Fungicidal mechanism of chlorine dioxide on *Saccharomyces cerevisiae* . Ann. Microbiol.y 63, 495–502. 10.1007/s13213-012-0494-8

