# Supplementary material for: Genome-wide identification of the PFK gene family and their expression analysis in Quercus rubra
Source: Front Genet. 2023 Nov 9;14:1289557. doi: 10.3389/fgene.2023.1289557 (PMC10665885; doi:10.3389/fgene.2023.1289557)

Supplementary Material

# Supplementary Table

Supplementary Table 1. Sequence of primers used for qPCR in of *Quercus rubra.*

| **Gene** | **Forward primer (5’→3’)** | **Reverse primer (5’→3’)** |
| --- | --- | --- |
| Qurub.02G097100.1 | ACCATCGTAAAGCCAGGGTG | TGGAAGACAAGTACCACGGC |
| Qurub.02G097100.2 | TGATTTGCTGGGACGGACAA | ACCTTCGTTGGGCATTTTGC |
| Qurub.02G189400.1 | TACAGGATTCACGGTTGGGC | TAGCACTCAAGAAGCTCGGC |
| Qurub.02G189400.2 | TACAGGATTCACGGTTGGGC | AGCACTCAAGAAGCTCGGC |
| Qurub.05G067500.1 | TCCAGATGAGATCGCGAAGC | ACACATCGGAATCGCTTGGT |
| Qurub.05G085700.1 | CGATATCGTTCTCCGCCACA | ACAGGTGACAATAGCAGCCC |
| Qurub.05G235500.1 | TACGTGCTGTTCCGAGCAAT | CTTGCCCACATCCTGTCAGT |
| Qurub.05G235500.2 | TACGTGCTGTTCCGAGCAAT | GGCGTGGCCTTAAAGTTGTG |
| Qurub.05G236300.1 | CACGAGGAGGCCATGATACC | CACAACTTTAAGGCCACGCC |
| Qurub.06G036700.1 | ATTGTTCGAGGCTGATCGCA | AAGAAGAGAAGTCGTGGCCG |
| Qurub.09G134300.1 | TCCCGAATTTCCCCAACTCG | TGGATAGGACGGCAAATCGG |
| Qurub.09G134300.2 | TCCCGAATTTCCCCAACTCG | TGGATAGGACGGCAAATCGG |
| Qurub.12G198800.1 | TGATTCAAGGCGAAGCTGGT | AACGTGCCATTAATGCTGCC |
| Qurub.12G198800.2 | TGATTCAAGGCGAAGCTGGT | GAAAGTGGACCCCTCTTGGG |
| *Qrα-tub* | GACGTGTCTGTGCTCTTGGA | AGCCCCATCAAATCTCAATG |
| *Qr18rRNA* | ATGCCGGCGACGCATCATT | CACTACCTCCCCATGTCAAGATTGGA |

Supplementary Table 2. Top 30 CREs detected from the promoters of *QrPFK* genes. The numbers under columns *QrPFK* and *QrPFP* indicate the total frequency distribution of CREs in *Q. rubra*.

| **Subcategory** | ***QrPFK*** | ***QrPFP*** | **Main category** |
| --- | --- | --- | --- |
| TATA-box | 395 | 119 | core promoter element around -30 of transcription start |
| CAAT-box | 294 | 77 | common *cis-*acting element in promoter and enhancer regions |
|  | 67 | 13 | short_function |
| Unnamed__4 | 57 | 11 |  |
| MYC | 37 | 6 |  |
| AT~TATA-box | 35 | 13 |  |
| Myb | 34 | 16 |  |
| ARE | 31 | 7 | *cis-*acting regulatory element essential for the anaerobic induction |
| Box 4 | 27 | 4 | part of a conserved DNA module involved in light responsiveness |
| ERE | 22 | 2 |  |
| MYB-like sequence | 14 | 1 |  |
| G-Box | 12 | 6 | *cis-*acting regulatory element involved in light responsiveness |
| ABRE | 12 | 6 | *cis-*acting element involved in the abscisic acid responsiveness |
| AAGAA-motif | 11 | 6 |  |
| TATA | 11 | 2 |  |
| TCT-motif | 9 | 2 | part of a light-responsive element |
| CGTCA-motif | 9 | 0 | *cis-*acting regulatory element involved in the MeJA-responsiveness |
| TGACG-motif | 9 | 0 | *cis-*acting regulatory element involved in the MeJA-responsiveness |
| STRE | 9 | 2 |  |
| as-1 | 9 | 0 |  |
| GT1-motif | 8 | 0 | light responsive element |
| Unnamed__1 | 8 | 1 | 60K protein binding site |
| Unnamed__1 | 8 | 1 |  |
| MRE | 7 | 1 | MYB binding site involved in light responsiveness |
| WRE3 | 6 | 2 |  |
| MBS | 5 | 3 | MYB binding site involved in drought-inducibility |
| W box | 5 | 3 |  |
| CCGTCC motif | 5 | 2 |  |
| CCGTCC-box | 5 | 2 |  |
| A-box | 5 | 2 | *cis-*acting regulatory element |

Supplementary Table 3. Pearson correlation analysis between protein and gene*.*

| **Gene** | **Correlation** | ***p*-value** |
| --- | --- | --- |
| Qurub.02G097100.1 | 0.7551** | 0.0045 |
| Qurub.02G097100.2 | 0.6436* | 0.0239 |
| Qurub.02G189400.1 | -0.7195** | 0.0083 |
| Qurub.02G189400.2 | -0.7046* | 0.0105 |
| Qurub.05G067500.1 | 0.6673* | 0.0178 |
| Qurub.05G085700.1 | 0.4366 | 0.1559 |
| Qurub.05G235500.1 | -0.0328 | 0.9193 |
| Qurub.05G235500.2 | -0.0438 | 0.8924 |
| Qurub.05G236300.1 | 0.2673 | 0.4010 |
| Qurub.06G036700.1 | 0.4814 | 0.1131 |
| Qurub.09G134300.1 | -0.75496** | 0.0045 |
| Qurub.09G134300.2 | -0.7272** | 0.0074 |
| Qurub.12G198800.1 | 0.3746 | 0.2302 |
| Qurub.12G198800.2 | 0.7271** | 0.0074 |

# Supplementary Figure

Supplementary Figure S1. Flow chart of methodology.


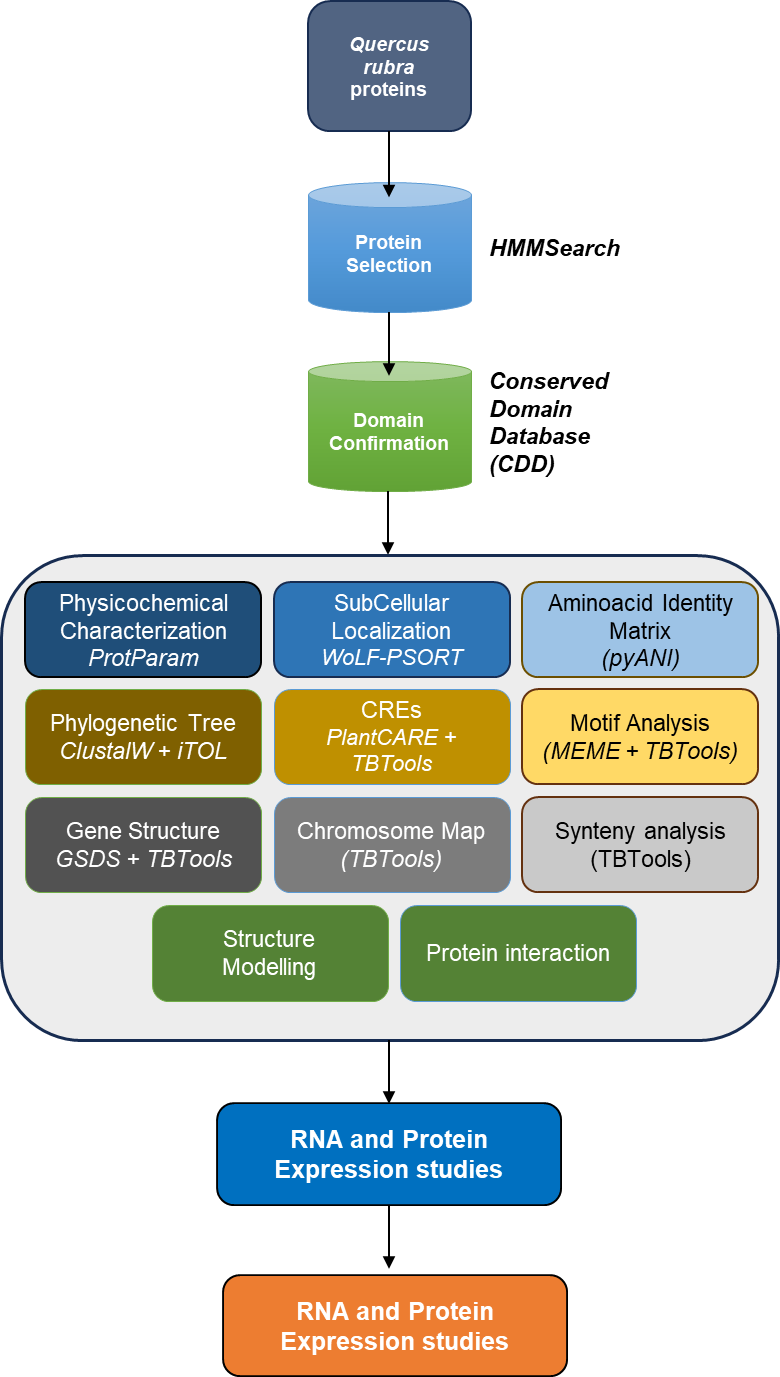


Supplementary Figure S2. Genetic identity matrix (%) derived from multiple alignments of amino acid sequences of proteins containing the PFK domain using ClustalW2


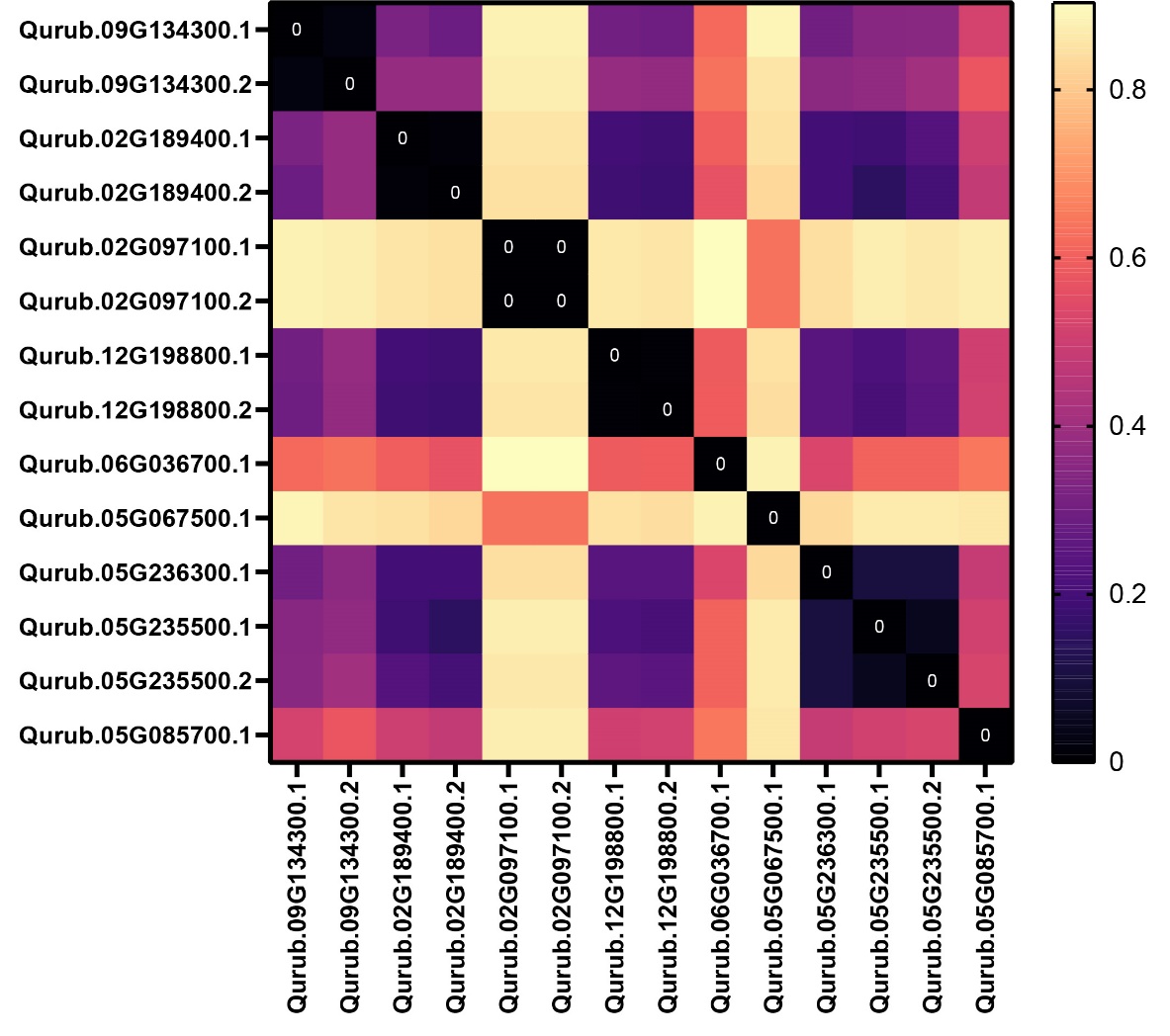


Supplementary Figure S3. Model validation using Ramachandran Plot of PFK family proteins. (a) Qurub.02G097100.1; (b) Qurub.02G097100.2; (c) Qurub.02G189400.1; (d) Qurub.02G189400.2; (e) Qurub.05G067500.1; (f) Qurub.05G085700.1; (g) Qurub.05G235500.1; (h) Qurub.05G235500.2; (i) Qurub.05G236300.1; (j) Qurub.06G036700.1; (k) Qurub.09G134300.1; (l) Qurub.09G134300.2; (m) Qurub.12G198800.1; (n) Qurub.12G198800.2


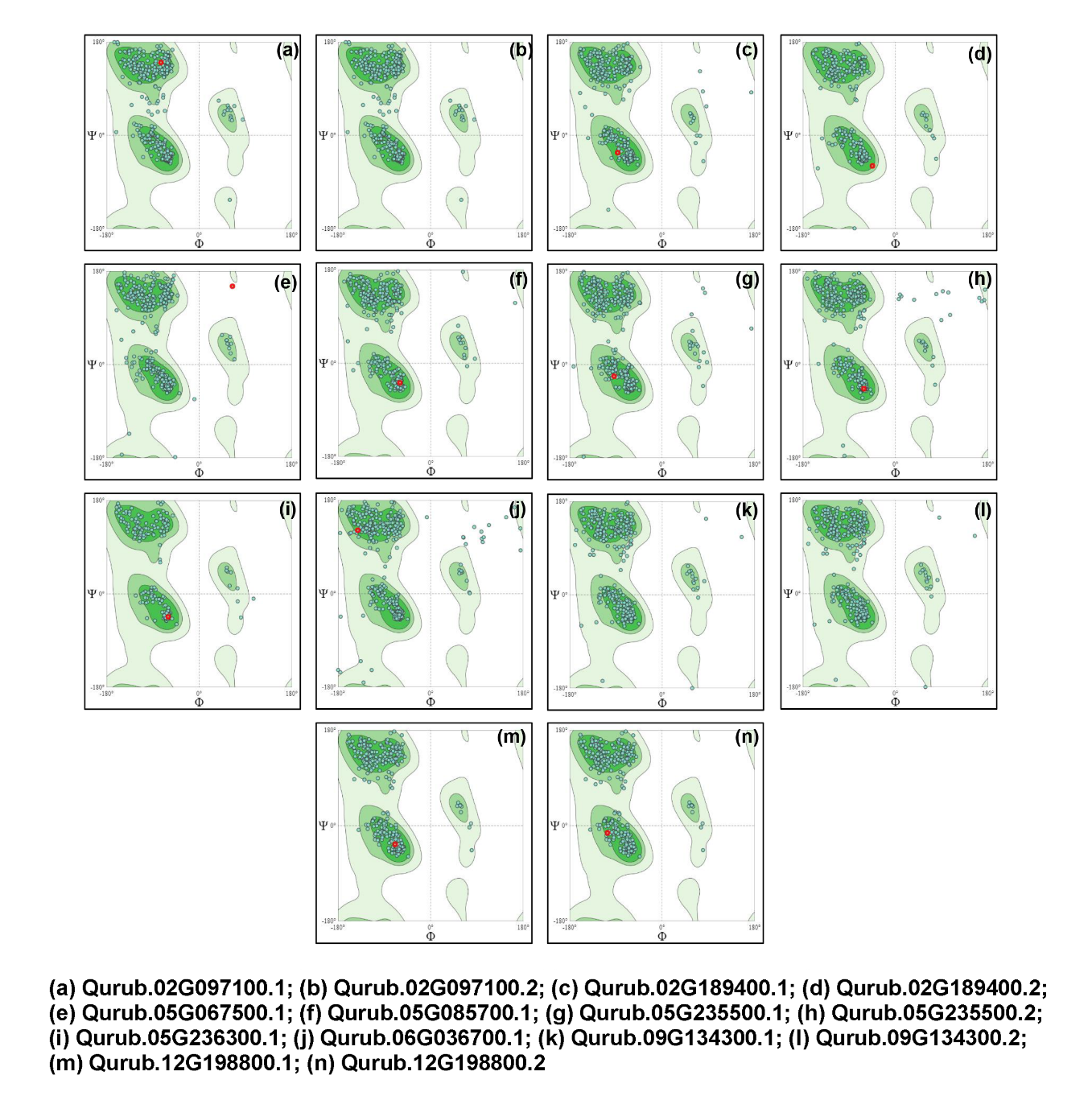

Supplement: Supplementary file 1 [file Table1.DOCX]
